# Cloning and molecular characterization of *Triticum aestivum* ornithine amino transferase (TaOAT) encoding genes

**DOI:** 10.1186/s12870-020-02396-2

**Published:** 2020-04-29

**Authors:** Alia Anwar, Maoyun She, Ke Wang, Xingguo Ye

**Affiliations:** 1 Institute of Crop Sciences, Chinese Academy of Agricultural Sciences, Beijing, 100081 People’s Republic of China; 2 School of Veterinary and Life Sciences, Murdoch University, Perth, WA 6150 Australia

**Keywords:** Wheat, Ornithine aminotransferase, Drought tolerance, Salt tolerance, Floret development

## Abstract

**Background:**

Ornithine aminotransferase (OAT, EC:2.6.1.13), alternatively known as ornithine delta aminotransferase (δOAT), is a pyridoxal phosphate (PLP)-dependent enzyme involved in the conversion of ornithine into glutamyl-5-semi-aldehyde (GSA) and vice versa. Up till now, there has been no study on OAT in wheat despite the success of its isolation from rice, maize, and sorghum. This study focuses on identification and molecular characterization of *OAT* in wheat.

**Results:**

In total, three homeologous *OAT* genes in wheat genome were found on chromosome group 5, named as *TaOAT-5AL*, *TaOAT-5BL*, and *TaOAT-5DL*. Sequence alignment between gDNA and its corresponding cDNA obtained a total of ten exons and nine introns. A phylogenetic tree was constructed and results indicated that OATs shared highly conserved domains between monocots and eudicots, which was further illustrated by using WebLogo to generate a sequence logo**.** Further subcellular localization analysis indicated that they functioned in mitochondria. Protein-protein interactions supported their role in proline biosynthesis through interactions with genes, such as delta 1-pyrroline-5-carboxylate synthetase (P5CS) and pyrroline-5-carboxylate reductase (P5CR), involved in the proline metabolic pathway. Promoter analysis exposed the presence of several stress responsive elements, implying their involvement in stress regulation. Expression profiling illustrated that *TaOAT* was highly induced in the wheat plants exposed to drought or salt stress condition. Upregulated expression of *TaOATs* was observed in stamens and at the heading stage. A potential role of *TaOAT* genes during floret development was also revealed. Furthermore, the transgenic plants overexpressing *TaOAT* showed enhanced tolerance to drought stress by increasing proline accumulation. In addition, salt tolerance of the transgenic plants was also enhanced.

**Conclusion:**

*TaOATs* genes were involved in proline synthesis and nitrogen remobilization because they interacted with genes related to proline biosynthesis enzymes and arginine catabolism. In addition, *TaOAT* genes had a role in abiotic stress tolerance and a potential role in floret development. The results of this study may propose future research in the improvement of wheat resistance to abiotic stresses.

## Background

Wheat (*Triticum aestivum*) is a worldwide cultivated crop and accounts for 20% of the calories consumed by humans [1]. However, adverse climate affects wheat productivity greatly, and at the same time rapid human population growth and arable land reduction exacerbate current shortages of wheat yield. Thus, improving wheat yield, especially under increasing abiotic stresses, is necessary to alleviate the situation. Plants have evolved a complex system to survive abiotic stresses via changes at the morphological, physiological and molecular levels [2, 3]. In the past decades, genetic engineering has made great progress in wheat breeding. Unfortunately, the limitations in the wheat germplasm (also known as wheat gene resources) has restricted the development of wheat varieties with abiotic stress tolerance. Therefore, it is necessary to identify more stress-related genes which can be utilized in breeding programs to develop stress-tolerant wheat varieties.

Ornithine aminotransferase is a highly conserved enzyme present in all prokaryotes and eukaryotes, from unicellular bacteria to multicellular animals and plants, which catalyze the transamination of ornithine (Orn) into glutamyl-5-semialdehyde (GSA) during proline (Pro) biosynthesis. This enzyme protein belongs to the aspartate aminotransferase (AAT) superfamily (fold type I) of pyridoxal phosphate (PLP)-dependent enzymes. The crucial role of the AAT superfamily is the control of nitrogen and carbon flux via linkage into the main pathways involved in carbon and nitrogen metabolism and protein biosynthesis [4]. The OAT enzyme functions in stress-induced proline accumulation in cytoplasm, programmed cell death and non-host disease resistance in plants through an alternative pathway known as the ornithine pathway [5, 6]. The OAT enzyme is also involved in nitrogen metabolism as evidenced by Pro metabolism showing positive correlation with nitrogen metabolism through two regulatory enzymes *i.e* OAT and P5CS, which is dependent on the nitrogen supply in plants [7]. Moreover, an acute role of OAT in arginine catabolism also confirms its function in nitrogen reutilization [8, 9].

The first plant *OAT* gene was cloned from *Vigna aconitifolia* by functional complementation of an *Escherichia coli* Pro auxotroph strain [10]. Subsequently, the *OAT* gene was isolated from *Arabidopsis* using a homologous cloning approach with transcripts found only in young seedlings in response to salt stress [11]. Due to the availability of the sequences in public databases, *OAT*-encoding genes have been successfully cloned and functionally characterized in a number of crops species. Involvement of OAT in drought and salinity stress has been reported in many plant species. For example, an increased OAT expression level was observed in cotyledons of NaCl-treated radish (*Raphanus sativus*) [12] and in young *Arabidopsis* plantlets exposed to 200 mM NaCl [11]. Similarly, overexpression of *OsOAT* enhanced the scavenging capacity of reactive oxygen species (ROS) under stressed conditions in rice [13]. However, most studies have been focused on the function of *OAT* genes under abiotic stresses based on model plants. No report has been conducted on staple crop species like wheat. Thus, *TaOAT*-encoding genes were isolated and functionally characterized from wheat in this study. Results of our molecular isolation and functional identification of *TaOAT*s under abiotic stresses can contribute to the improvement of stress tolerance breeding in wheat.

## Results

### Isolation and structure analysis of *TaOAT* genes in hexaploid wheat

**Fig. 1:**
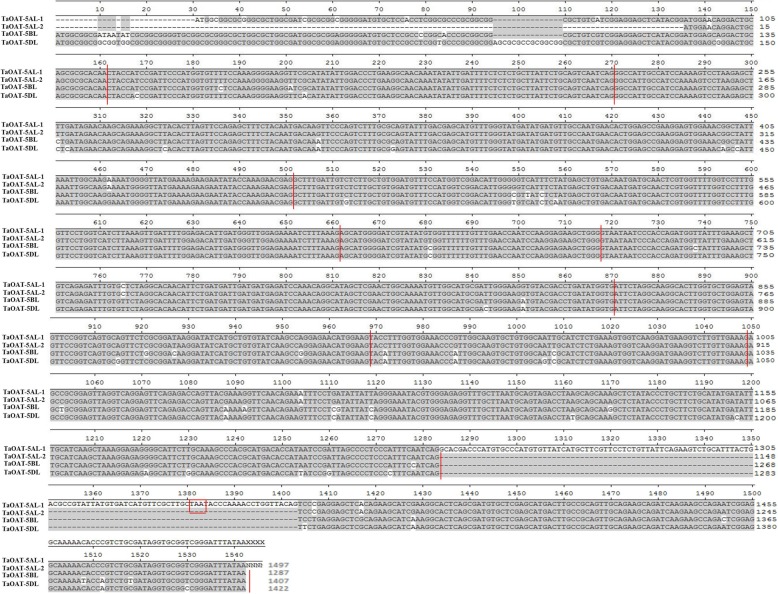
Alignment of the cDNAs sequences of *TaOAT* gene*s* amplified from the hexaploid wheat cultivar Fielder. In total there are four alleles named *TaOAT-5AL-1*, *TaOAT-5AL-2*, *TaOAT-5BL*, and *TaOAT-5DL* with sizes of 1497 bp, 1287 bp, 1407 bp, and 1422 bp, respectively. The *TaOAT-5AL-1* contained in-frame stop codon indicated by red box that caused the incomplete translation of protein. The *TaOAT-5AL-2*, *TaOAT-5BL*, and *TaOAT-5DL* showed complete translation of proteins. Intron-exon junction is divided by red lines. Dotted lines indicate the deletion

Sequence retrieval from the International Wheat Genome Sequencing Consortium (IWGSC) database using *AtOAT* accession At5g46180 as query resulted in a total of three scaffolds that matched our query, namely, TGACv1_scaffold_374190, TGACv1_scaffold_404925, and TGACv1_scaffold_435304, which were located on the long arm of chromosome group 5 with e-values of 1e-30, 7e-27 and 4e-24, respectively. Predictions of the open reading frame (ORF) of the three candidate genes’ gDNA/cDNA lengths were 4568/1419 bp, 4276/1488 bp, and 4446/1422 bp, respectively. No variation was found between the sequence of the common wheat cultivar Fielder and the reference sequence of Chinese Spring. However, two transcript variants of *TaOAT-5AL* were revealed. These were named *TaOAT-5AL-1* and *TaOAT-5AL-2* and characterized by 1497 bp and 1287 bp in cDNA length, respectively. Compared to *TaOAT-5AL-2*, *TaOAT-5AL-1* contained an additional 120-bp insertion encompassing an in-frame stop codon, which resulted in a premature protein (Fig. 1). The additional insertion was genotypically confirmed by sequencing results from six cultivars used in this study. *TaOAT-5AL-2* was identical to the reference sequence based on the sequencing results. Interestingly, there were six splice variants in the *T. dicoccoides* database and the *TaOAT-5AL-2* transcripts showed high similarity to two of these variants, TRIDC5AG054810.2 and TRIDC5AG054810.3 (Additional file 1: Figure S1). After sequence alignment, we found that *TaOAT-5AL-2* had higher identity to TRIDC5AG054810.2. We suspect that during the evolution of hexaploid wheat, it retained two alternative-spliced variants for *TaOAT-5AL*. On the other hand, only one transcript has been found for *TaOAT-5BL* and one for *TaOAT-5DL*. In addition, *TaOAT-5BL* had 1407 bp instead of 1488 bp due to an 81-bp deletion, which is the same as the putative transcript in the ensemble Plants database with an accession ID of TraesCS5B02G376900.1. There was no difference in bp and length for *TaOAT-5DL* transcript. We have submitted these sequences to the National Center for Biotechnology Information (NCBI) database and their accession numbers are MK942062, MK942063, MK680533 and MK748213 for *TaOAT-5AL-1*, *TaOAT-5AL-2*, *TaOAT-5BL*, and *TaOAT-5DL*, respectively.

**Fig. 2:**
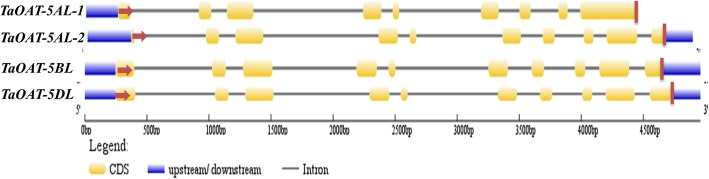
Structure of *TaOAT* genes in common wheat. The transcripts viz *TaOAT-5AL-2*, *TaOAT-5BL*, and *TaOAT-5DL* show ten exons and nine introns while transcript viz *TaOAT5AL-1* shows the disrupted gene structure due to the retention of the 9th intron that fused with the 9th exon causing the larger 9th exon as compared to the other transcripts. Gray, yellow and blue colors respectively represent introns, exon and UTRs. The red arrow and line show the start (ATG) and stop codon (TAA), respectively

The exon-intron boundaries were determined by comparing the gDNA sequence in each genome with the full-length cDNA sequences of *TaOAT-5AL-1*, *TaOAT-5AL-2*, *TaOAT-5BL*, and *TaOAT-5DL*. In total, the *TaOAT* gene contained ten exons and nine introns. The *TaOAT-5AL* gene had two types of transcripts which were likely a consequence of alternative splicing. *TaOAT-5AL-1* consisted of nine exons due to the retention of the 9th intron, resulting in the formation of a premature protein (Fig. 2). Sequence analysis showed a nucleotide transition (T → C) occurred in the gDNA of *TaOAT-5AL* (the transition is indicated by the red box in Additional file 1: Figure S2), which breaks the classic boundary of intron splicing (5′-GT(N)nAG-3′; where N represents any nucleotide and n represents a random number). The transition likely caused the retention of the 9th intron in *TaOAT-5AL-1* corresponding to that in *TaOAT-5BL* and *TaOAT-5DL* (Fig. 2). However, *TaOAT-5AL-2* has the same structure as *TaOAT-5BL* and *TaOAT-5DL* (Fig. 1). The three genes have 87.68 and 87.18% identity at the gDNA and cDNA levels, respectively.

### Chromosomal and subcellular localization of *TaOAT* genes and their encoding proteins in wheat

**Fig. 3:**
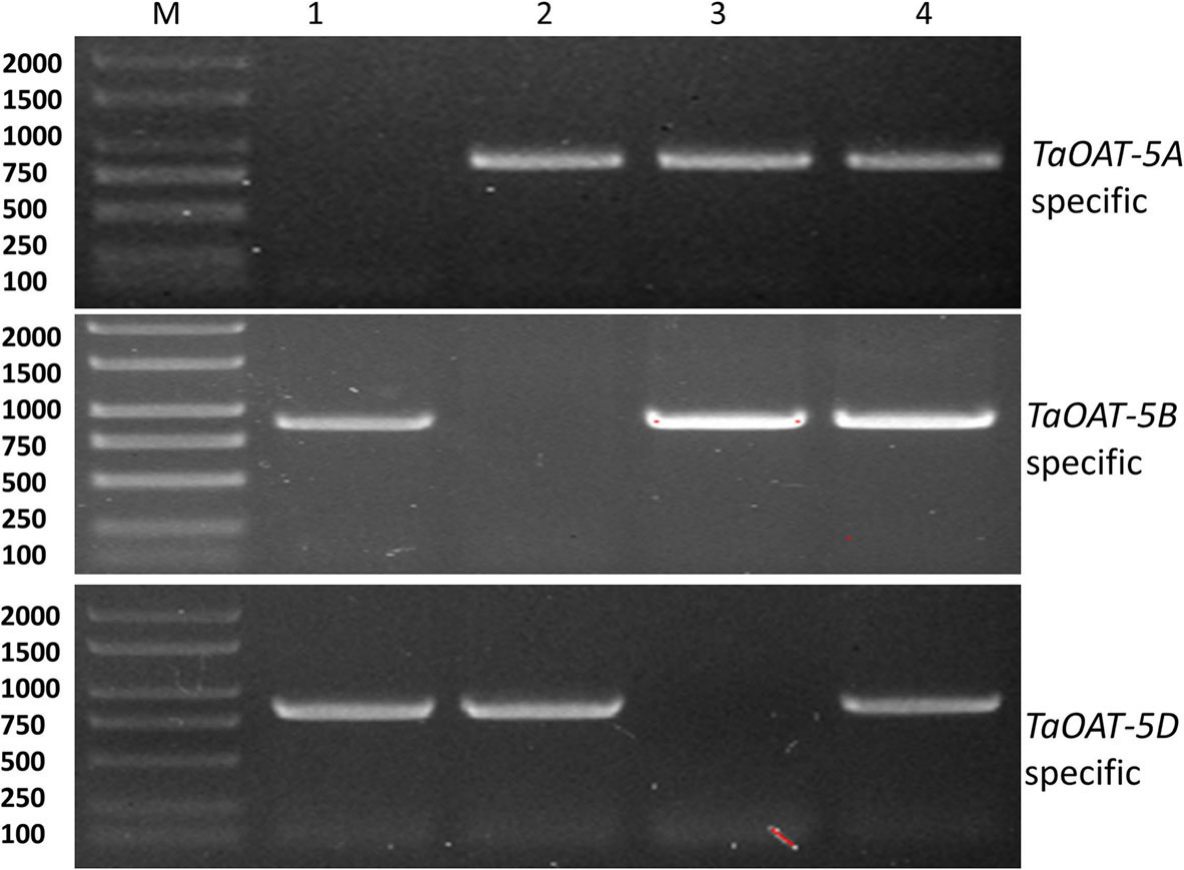
Determination of chromosomal location of *TaOAT* genes in hexaploid wheat using specific primers and nullitetrasomic lines. The absence of the band 864 bp in size in lane 1 shows that the primer is specific to *TaOAT-5AL* due to the absence of chromosome 5A in N5A/T5B. Similarly, the absence of the bands 882 and 840 bp in size in lane 2 and 3 shows the specificity of the primers to *TaOAT-5BL* and *TaOAT-5DL* as chromosomes 5B and 5D are absent in N5B/T5A and N5D/T5A lines, respectively. M: DL2000 DNA ladder (TianGen Biotech. Beijing Co., Ltd.); 1–4: Chinese Spring chromosome group 5 nullitetrasomic lines where N represents the null chromosome, and T represents the tetra chromosome in which 2 chromosomes replaced or shuffled the respective absent two chromosomes (1: N5A/T5B, 2: N5B/T5A, 3: N5D/T5A, and 4: Chinese Spring)

To confirm the chromosomal location of *TaOAT* genes in wheat, primers specific to each *TaOAT* gene were designed and the localization was performed by using three Chinese Spring nullitetrasomic lines related to chromosome group 5 as templates for the PCR assay in which Chinese Spring (CS) was used as a control. No band was obtained in lane 1 for N5A/T5B when using the specific primers of *TaOAT-5AL*, in lane 2 for N5B/T5A when using the specific primers of *TaOAT-5BL*, and in lane 3 for N5D/T5A when using the specific primers of *TaOAT-5DL* (Fig. 3). The absence of these bands suggests a deletion of the gene due to the corresponding chromosome removal. Therefore, the three genes, *TaOAT-5AL*, *TaOAT-5BL*, and *TaOAT-5DL,* were experimentally assigned to chromosome 5A, 5B, and 5D, respectively, in hexaploid wheat.

**Fig. 4:**
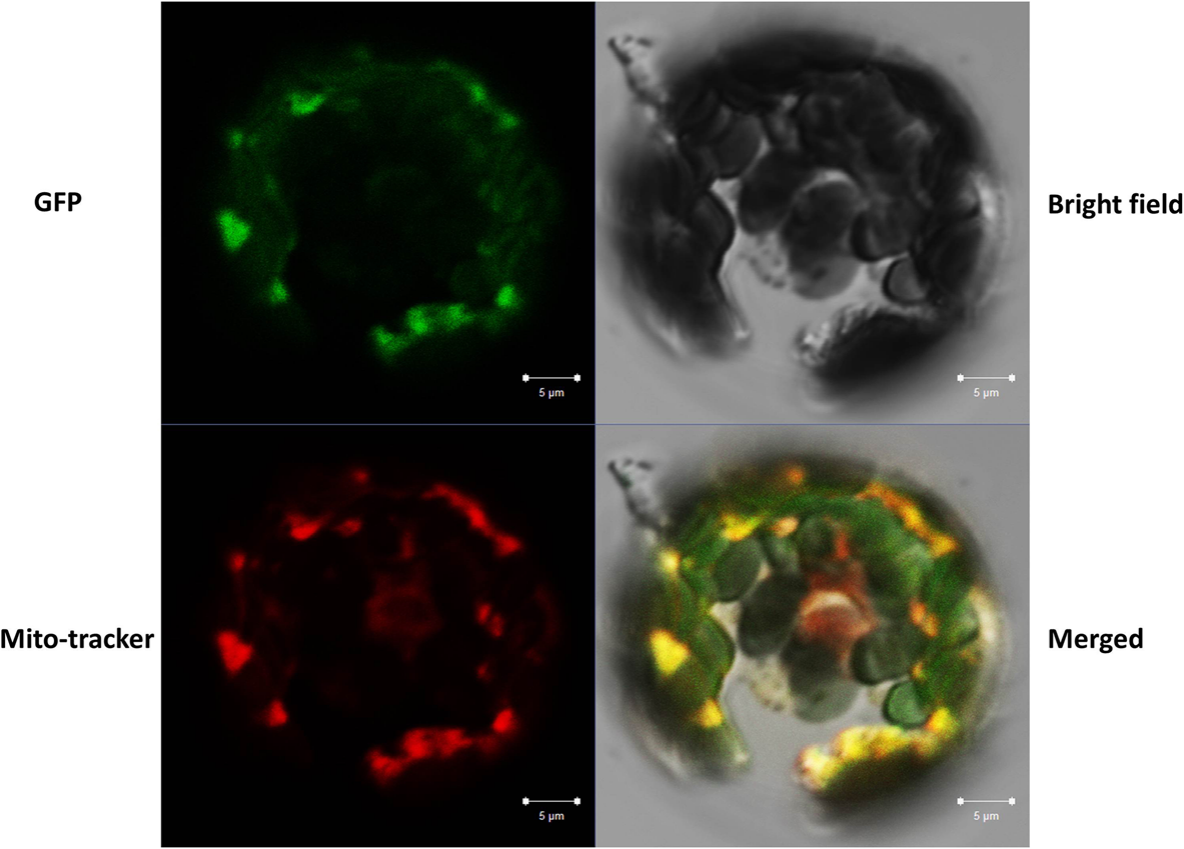
Subcellular localization of TaOAT protein. Green fluorescence indicates the GFP signals from TaOAT C terminal fused GFP vector, red indicates mitochondria stained with mitotracker dye, and yellow shows the merged signals of GFP and mitotracker. The size for scale bar is 5 μm

Subcellular localization of plant OAT proteins of 65 species was predicted using TargetP. Most of the plant OATs (83%) were targeted to the mitochondria (Additional file 2: Table S1). Because OATs are thought to be highly conserved enzymes and previous reports have shown that AtOAT and OsOAT are targeted to mitochondria [8, 9], we speculated that TaOAT proteins also functions in mitochondria, which was supported by the high probability of 0.9707 obtained using Mitoprot in this study. Moreover, the transient expression of TaOAT-fused GFP signals was only observed in the mitochondria of wheat protoplasts; the merging of mitochondrion-specific dye with GFP signals indicated mitochondrion-targeting of TaOAT proteins (Fig. 4).

### Phylogenetic and promoter analysis of wheat *OAT* genes

The phylogenetic analysis showed that there were two distinguished groups, one among the monocots (indicated by red) and the other among the eudicots (indicated by blue) (Additional file 1: Figure S3). The OATs from cereals like maize, sorghum, and rice formed a distinguished but small cluster and shared high similarity. The homologous genes in wheat showed a close similarity with its respective ancestral group, for example *TaOAT-5AL* showed high similarity with *Triticum urartu* and *TaOAT-5DL* showed high similarity with *Aegilops tauschii*, implying that gene structure and function were shared by common wheat and its wild relatives. Overall, these results suggest that OAT from common wheat and its wild relatives remained highly conserved during evolution. In contrast, the larger group of dicot *OAT* genes formed five sub-groups, indicating that dicot *OAT* genes have evolved more diverse functions. The conservation of the targeted genes among monocot and dicot species was also illustrated in a sequence logo created using WebLogo (Additional file 1: Figure S4).

In this study, 1000 base pairs upstream of the start condon of *TaOATs* were selected for the prediction of *cis*-elements using an online tool. The results showed that many of the predicted elements were stress-responsive, including abscisic acid responsive element (ABRE), MYB (Myeloblastosis) *cis*-elements, ROS-related motifs (G-box and W-box), ethylene-responsive element (ERE), heat shock element (HSE), APETALA2-like (AP-2-like) element and low temperature responsive (LTR) element (Additional file 1: Figure S5). The G-box (CACGTG) element is involved in responses to light, abscisic acid, methyl-jasmonate and anaerobiosis. The G-box also has a role in ethylene induction as well as in seed-specific expression. Additionally, the G-box also functions as an ABRE (ABA-responsive element) [14, 15]. Both ABRE and G-box elements provide the binding sites for bZIP transcription factors (TFs) that regulate stress responses. Both ABRE and G-box have been shown to be present in the three wheat *OAT* genes of this study. The W box is present in *TaAOT-5AL* and *TaOAT-5DL* which interacts with TFs belonging to the WRKY family. The ABRE present in all *TaOAT* genes, DREs (dehydration responsive elements) in *TaOAT-5AL* and *TaOAT-5DL*, and LTR in *TaOAT-5DL* provide the binding sites for *NAC* genes, implying TaOATs role in both salinity and drought stress response. In addition, the AP-2-like domain has been found in *TaOAT-5DL*, which supports its role in floret development. These findings suggest that *TaOATs* have potential roles in plant responses to drought, salinity and pathogen stresses.

### Protein-protein interactions realted to wheat OAT

**Fig. 5:**
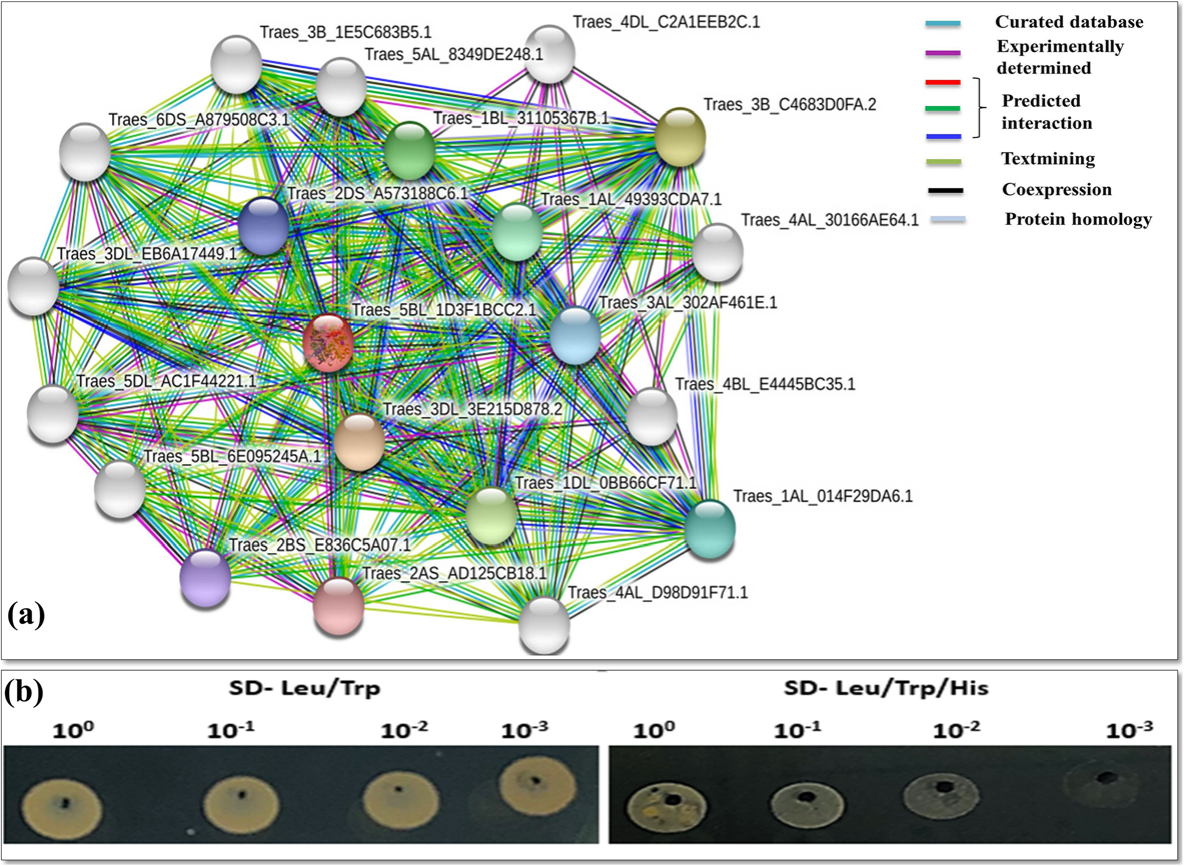
Protein-protein interaction of TaOAT with other wheat proteins. **a** The interacting protein partners predited by STRING database. The *TaOAT-5BL* (Traes_5BL_1D3F1BCC2.1) amino acid sequence was used to search for its interacting genes in wheat. Nodes represent the single protein coding locus and edges represent the meaningful interaction that shared the same functions. Color nodes represent the first shell of interactors while white nodes represent the second shell of interactors. Empty nodes represent the protein of unknown 3D structure while filled nodes represent the known or predicted 3D structure. **b** Confirmation of the predicted interaction results by the yeast two hybrid. The figure on the left hand side represents the yeast growth on two absent (Leu/Trp) medium. The figure on the right hand side represents the yeast growth on three absent (Leu/Trp/His) medium. The specific interaction was determined by the growth of transformants on three absent (Leu/Trp/His) medium. 10^0^ represent the original culture sample. 10^− 1^–10^− 3^ showed 10 fold serial dilution of the sample

According to the STRING database, TaOAT interacts with Traes_1BL_31105367B.1 (delta 1-pyrroline-5-carboxylate synthetase), Traes_2BS_E836C5A07.1 (an uncharacterized protein in the arginase family), Traes_3B_1E5C683B5.1 (pyrroline-5-carboxylate reductase that belongs to the pyrroline-5-carboxylate reductase family), Traes_3DL_EB6A17449.1 (pyrroline-5-carboxylate reductase), Traes_4BL_E4445BC35.1 (a regulatory subunit of cyclin-dependent kinases), Traes_5BL_6E095245A.1 (arginine decarboxylase belonging to the Orn/Lys/Arg decarboxylase class-II family, SpeA subfamily), and many uncharacterized proteins (Fig. 5a, Additional file 3). Interestingly, Traes_2BS_E836C5A07.1 is wheat arginase (*TaARG*) gene which have been functionally characterized in our previous publication [16]. In this study, TaOAT-5BL protein showed the interaction with 2BS_E836C5A07.1 (TaARG) by STRING database and this interaction was also experimentally tested by yeast two hybrid assay (Fig. 5b) which demonstrated the positive interaction of both genes. These results also supported the predicted interaction. The interaction of TaOAT with P5CS and P5CR supports the role of TaOAT in proline biosynthesis. Additionally, the interaction of TaOAT-5BL with the proteins in the arginase family (TaARG-2BS) implies the involvement of TaOAT in arginine metabolism (Fig. 5b; Additional file 1: Figure S6).

### Expression profile of the wheat *OAT* gene in different tissues and developmental stages

**Fig. 6:**
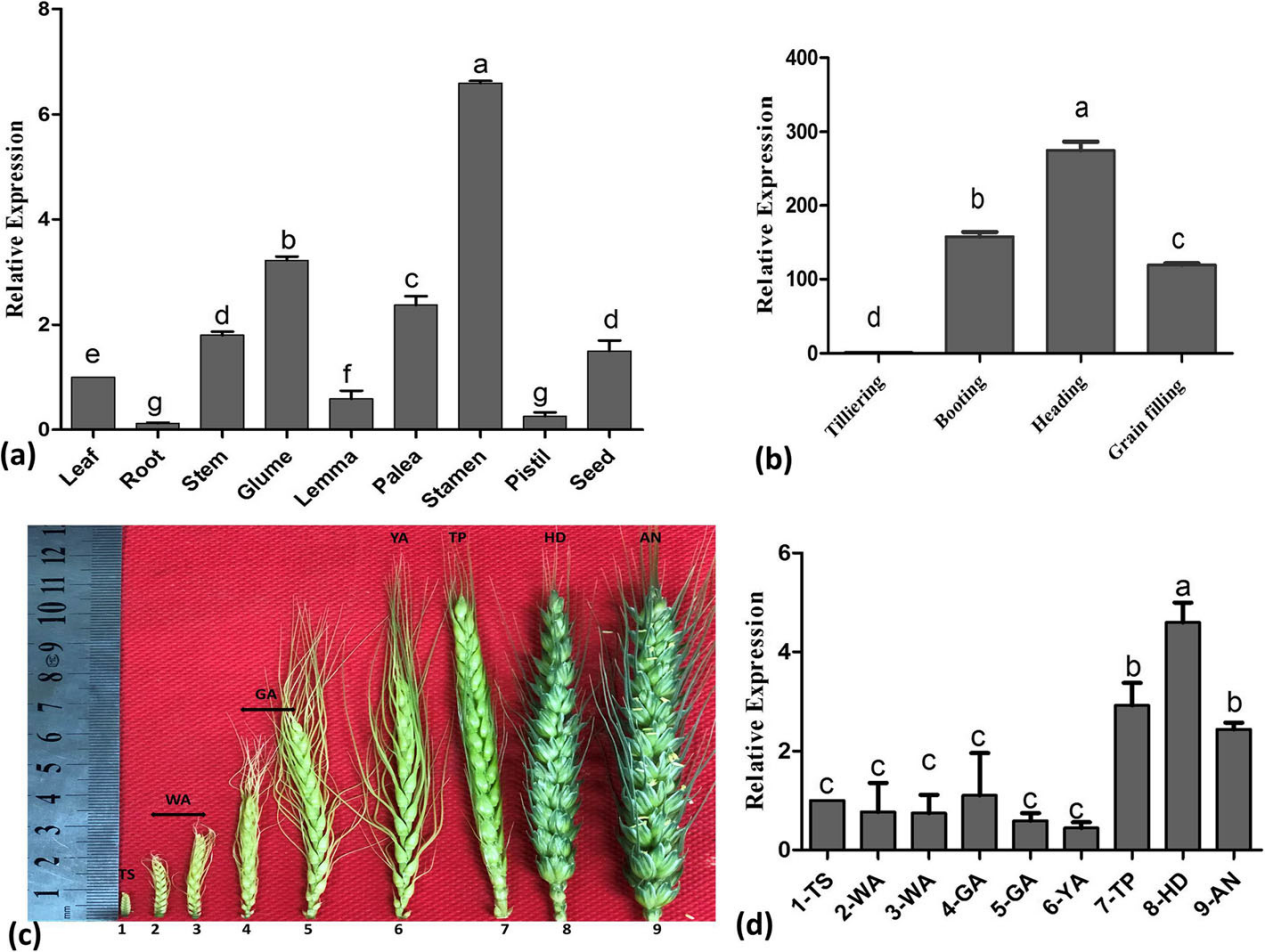
Expression profiles of wheat OAT genes in different tissues and developmental stages. **a** Expression pattern of *TaOAT* in different tissues. **b** Expression pattern of *TaOAT* at different developmental stages. **c** The seven spikelet-developmental stages according to Kirby and Appleyard (1987). The stages, from left to right, are: terminal spikelet stage (TS), white anther stage (WA), green anther stage (GA), yellow anther stag (YA), tipping stage (TP), heading stage (HD), and anthesis stage (AN). **d** Relative expression at the seven floret-developmental stages. Three biological replicates were averaged and the statistical analysis was performed using IBM-SPSS statistic 20. Small letters in bold represent significant differences between groups at α < 0.05 using Duncan’s multiple range tests. Bars indicate the standard error of the mean

To investigate the expression patterns of *TaOATs*, quantitative reverse transcription PCR (qRT-PCR) was performed in different tissues of wheat line Fielder. High *TaOAT* transcript levels were observed in stamens; moderate expression occurred in the leaf, seed, stem, and glume; and very low expression occurred in the root, pistil and palea (Fig. 6a). The expression pattern of *TaOAT* is similar to those of *OsOAT* [8]. The relative expression of transcripts in leaves gradually upregulated until it peaked at the heading stage and then decreased at the grain filling stage (Fig. 6b). Results of OAT expression at spike-developmental stages were strong at tipping, heading, and anthesis stages with the highest expression at the heading stage (Fig. 6c, d). The high expression observed in the stamen and low expression observed at the anthesis stage suggest that *TaOATs* are likely to be involved in anther dehiscence.

### Expression patterns of *TaOAT* genes induced by exogenous PEG and NaCl

**Fig. 7:**
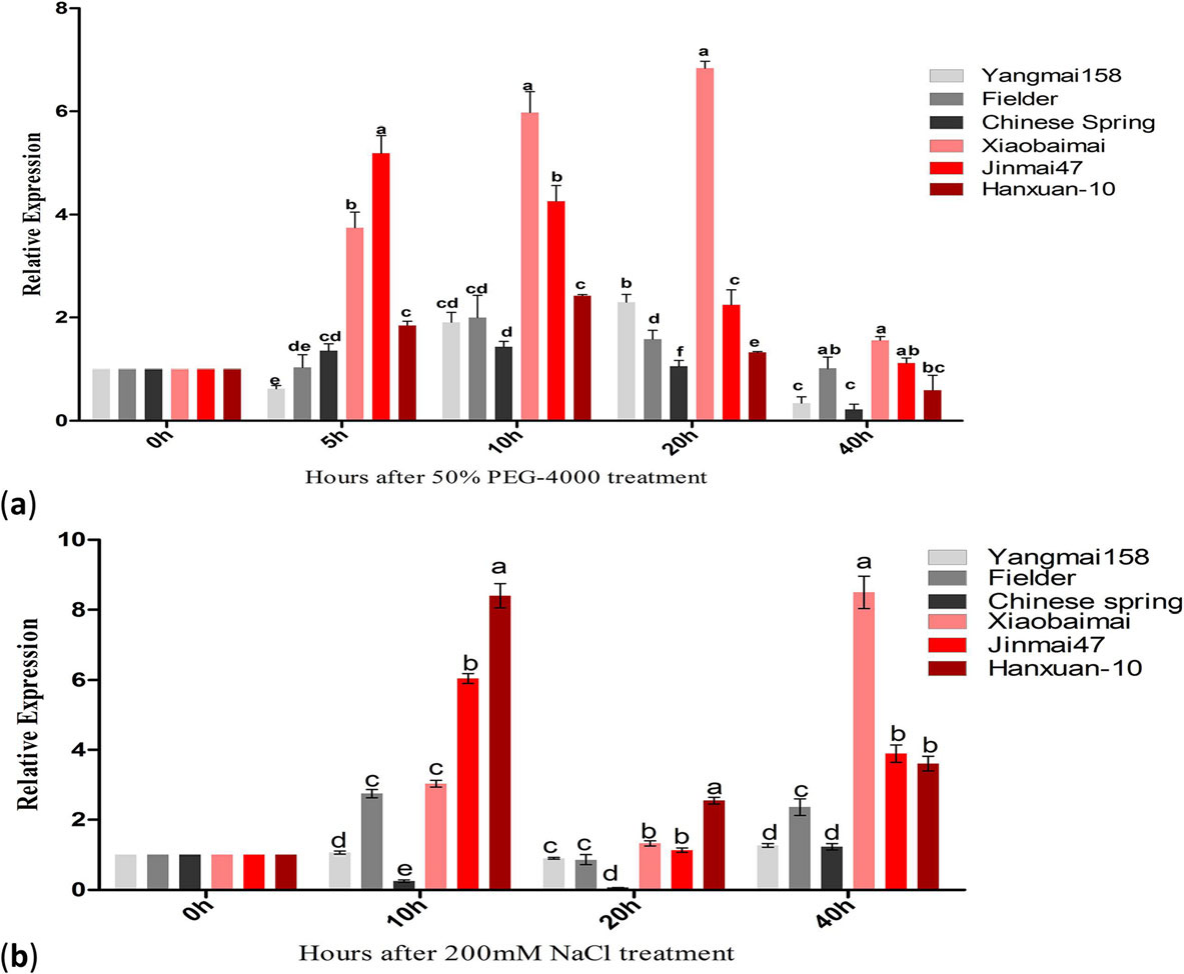
Expression pattern analyses by qRT-PCR of *TaOAT* genes upon PEG and NaCl treatment. **a** Transcript levels of *TaOAT* genes in wheat seedlings exposed to 50% PEG-4000 for different time periods. **b** Transcript levels of *TaOAT* genes in wheat seedling exposed to 200 mM NaCl stress for different time periods. Three biological replicates were averaged and statistical analysis was performed using IBM-SPSS statistic 20. Small letters in bold represent significant differences between groups at α < 0.05 using Duncan’s multiple range tests. Bars indicate the standard error of the mean

To refine the influence of abiotic stresses (drought and salt stresses) on expression of *TaOATs*, three drought-tolerant cultivars and three drought-susceptible cultivars were utilized and their expression levels of *TaOATs* were compared. The expression level at 0 h was set as the reference for comparisons in the data analysis (Fig. 7). The expression trends of *TaOATs* under PEG and NaCl stresses were similar despite their different responses in different wheat cultivars. For example, upregulated expression of *TaOATs* was more obvious in drought tolerant wheat cultivars than that in drought susceptible ones. The general expression trend of *TaOATs* first increased then decreased over time in both stress treatments of PEG and NaCl exposure. Expression peaked once at 10 h and another time at 20 h due to the 50% PEG-4000 treatment and then decreased at 40 h (Fig. 7a). Similarly, peaks of expression were observed at 20 h and 40 h due to the exposure to 200 mM NaCl stress (Fig. 7b). These results clearly suggested that *TaOATs* play a significant role in drought and salt stress.

### Generation of stable transgenic lines and drought tolerance test

**Fig. 8:**
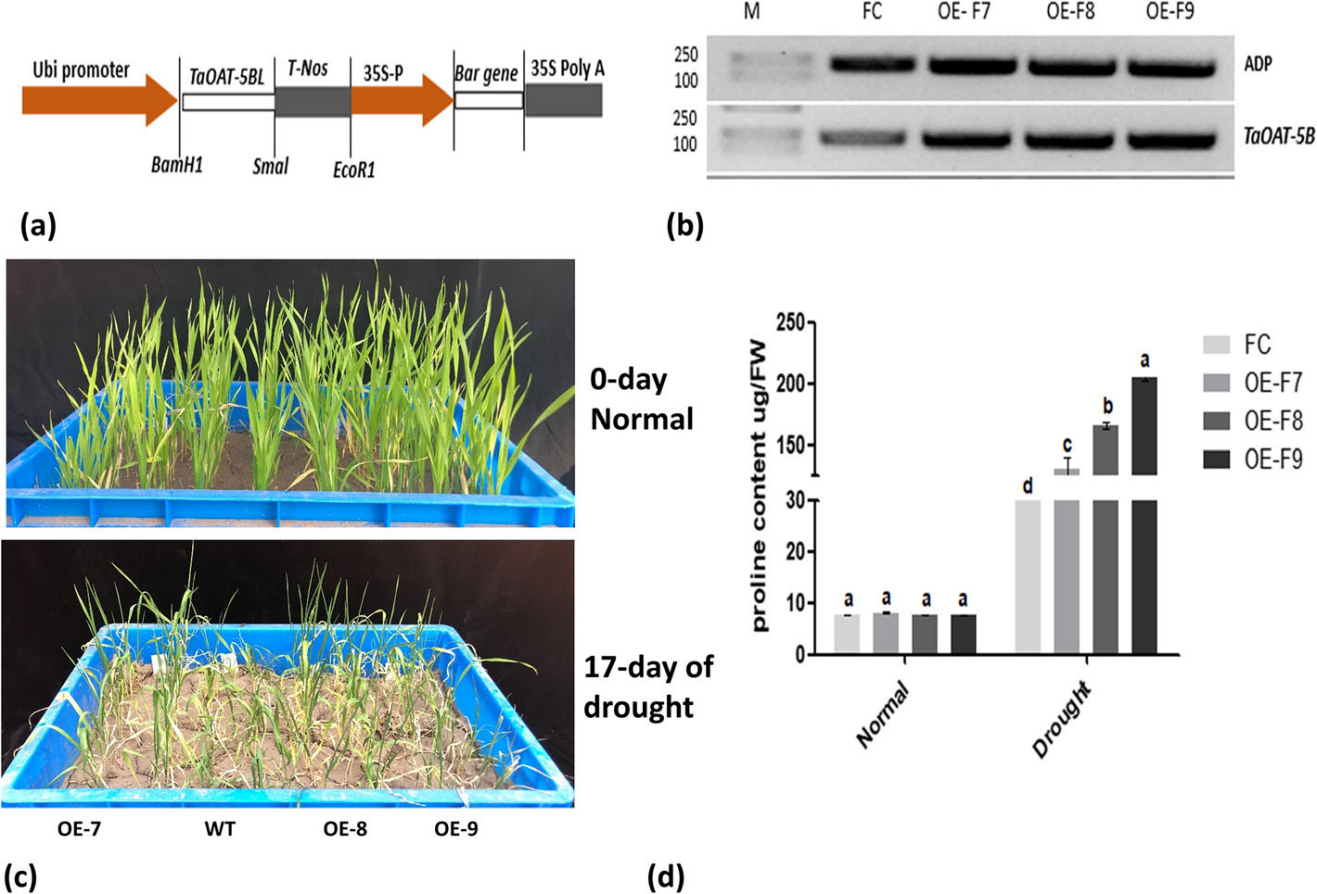
Vector construction and drought tolrenace test of *TaOAT-5BL* overexpressing transgenic wheat lines. **a** Schematic structure of expression vector used for *Agrobacterium*-mediated transformation. **b** Semi-quantitative PCR profiles for *TaOAT-5BL* in transgenic lines and their wild type Fielder. **c** Phenotype of *TaOAT-5BL* transgenic lines and wild type Fielder before and after drought stress. **d** Free proline content measured in the transgenic lines and wild type Fielder before and after drought stress. FC represents wild type Fielder and OE-F7, OE-F8, and OE-F9 are *TaOAT-5BL* overexpressing transgenic lines derived from Fielder. Data was analyzed using IBM-SPSS statistic 20. Small letters in bold represent significant differences between groups at α < 0.05 using Duncan’s multiple range tests. Bars indicate the standard error of the mean

Totally, 35 independent transgenic wheat plants were obtained by *Agrobacterium*-mediated transformation, among which 30 were found to be positive with transgenes *TaOAT-5BL* by PCR detection and *bar* by a QuickStix Kit (Additional file 1: Figure S7). Six stable independent transgenic lines were obtained in T_2_ generation and 3 of them named as OE-F7, OE-F8, and OE-F9 were used to perform functional analysis. Semi-quantitative PCR analysis demonstrated that the transgenic lines showed significantly higher expression of *TaOAT-5BL* than wild type Fielder (Fig. 8b).

The transgenic lines were subjected to water withholding at three-leaf-stage to test the contribution of *TaOAT-5BL* on drought tolerance. Seventeen days after water stress, wild type Fielder was severly effected in growth by drought as compared to its corresponding transgenic lines (Fig. 8c). As OAT is predicted to be involved in proline biosynthesis, free proline content was measured at normal and stress conditions. The results showed that there is no difference in proline content between transgenic plants and the wild type under normal condition; under drought stress condition, the transgenic plants accumulated more proline than the wild type plants. These results suggested the involvevment of *TaOAT-5BL* in proline biosynthesis under drought stress condition.

### Transgenic plants showed enhanced tolerance to salt stress in vitro condition

**Fig. 9:**
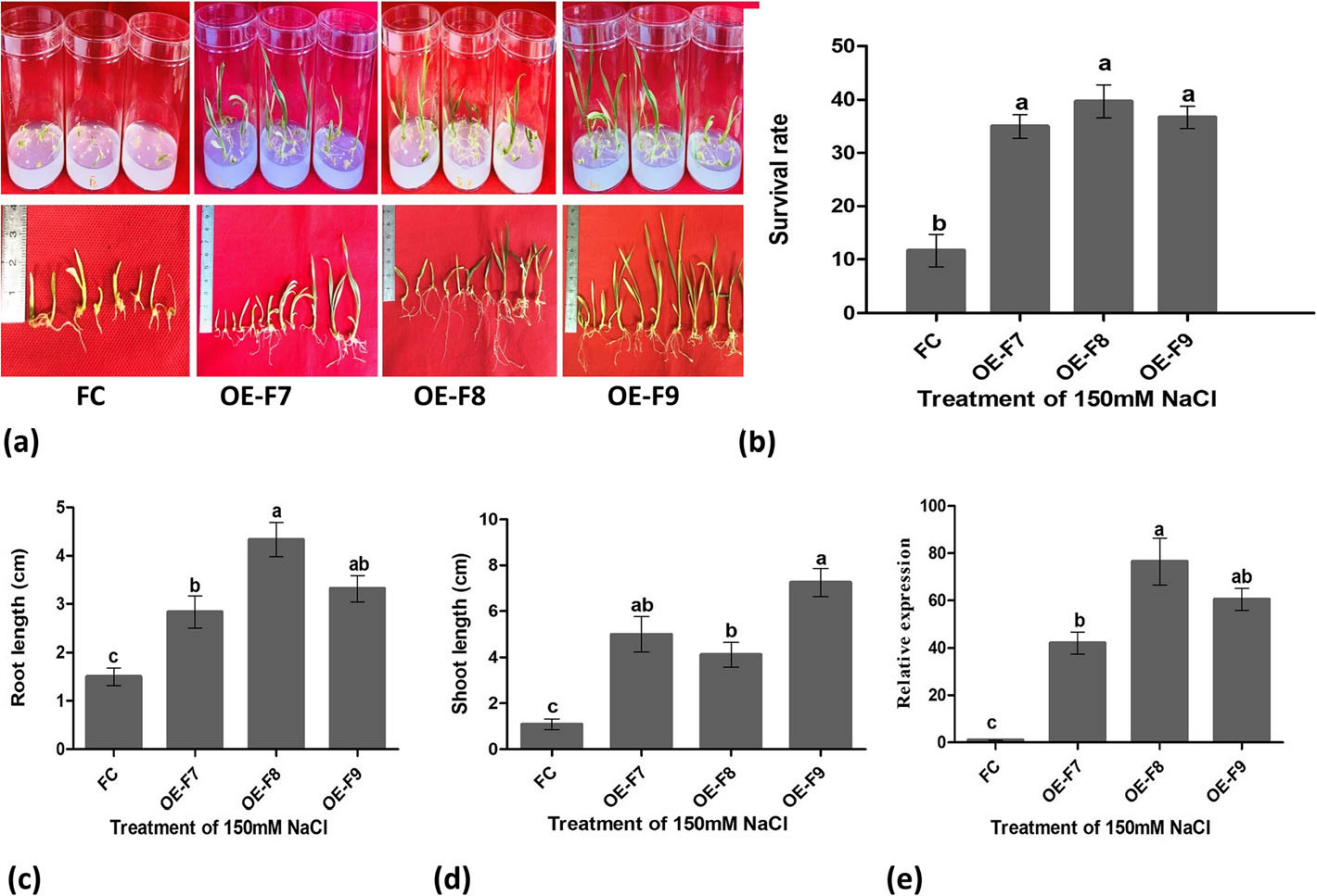
Salt tolerance test of *TaOAT-5BL* overexpressing transgenic lines and their wild type Fielder in 150 mM NaCI containing medium. **a** Salt tolerance phenotype of *TaOAT-5BL* transgenic lines and wild type Fielder cultured for 30 days. **b** Survival rate of *TaOAT-5BL* transgenic lines and wild type Fielder cultured 30 days. **c** Relative expression analysis of *TaOAT-5BL* in transgenic lines and wild type Fielder cultured for 30 days. **d** Root length of the surviving plants cultured for 30 days. **e** Shoot length of the surviving plants cultured for 30 days. FC represents wild type Fielder and OE-F7, OE-F8, and OE-F9 are *TaOAT-5BL* overexpressing transgenic lines derived from Fielder. The data was average of three replications and analyzed using IBM-SPSS statistic 20. Small letters in bold represent significant differences between groups at α < 0.05 using Duncan’s multiple range tests. Bars indicate the standard error of the mean

On 150 mM salt containing medium, the mature embryos of transgenic plants germinated with a rate of 71–85% while the mature embryos of the wild type germinated only with a rate of 27% (Additional file 1: Figure S8). Thirty days after inoculation on the salt medium, survival rate was 35–40% for the transgenic plants and 12% for the wild type plants (Fig. 9a, b). Additionally, the trangenic plants showed faster growth, longer and denser roots than the wild type plants (Fig. 9c, d). Relative expression analysis demonstrated that *TaOAT-5BL* was greatly up-regulated in the transgenic plants as compare to the wild type plants in response to salt stress condition (Fig. 9e). These results clearly depicted that *TaOAT-5BL* ehnanced salt tolerance of transgenic plants due to its high expression.

## Discussion

Drought and salt stresses can be critical limiting factors in plant growth and development that drastically impact crop productivity. As a result of global climate change, the rising temperatures will likely lead to severe episodes of drought and thus reduce the productivity of field crops. To prevent the potential global disaster of food shortage in the near future, demands for new cultivars of crops with improved genetic backgrounds are increasing. Therefore, it is of utmost importance to develop such cultivars which are more resistant to both salt and drought stresses without negatively impacting the yield of the crop. Towards this end, there is an urgent need to identify more genes that are responsible for drought and salt tolerances.

To date, many stress responsive genes have been identified and are grouped into two categories. The first category includes functional genes encoding metabolic enzymes related to osmo-protection, such as Pro metabolic enzymes, and the second category includes genes functioning in regulation, known as TFs [17]. The *OAT* gene is among the Pro metabolic enzymes which have been identified in a number of crop species [11, 13, 18, 19]. Until now, there is no report on cloning and functional characterization of the *OAT* gene in wheat. In this study, wheat *OATs* have been successfully cloned and their potential functional roles have been reported. Besides their involvement in Pro metabolism, it has commonly been thought that *OAT* functions in an alternative pathway for drought- and salt-stress-induced Pro accumulation [20, 21]. A 200-mM NaCl treatment can induce *OAT* expression in radish cotyledons and in *Arabidopsis* seedlings [11, 12, 22]. Similarly, *OsOAT* overexpression showed significantly increased tolerance to oxidative stress in rice [13]. To determine its function in wheat, in silico analysis of *TaOAT* gene promoter regions was initially performed. Several putative TF-binding sites have been found in the promoter of *TaOAT* genes, such as ABRE, MYB, ERE LTR, DRE, W-box, G-box, and HSE, which provide the binding sites for bZIP, WRKY, and NAC TFs. Several reports associate these TFs with plant responses to drought and salt stresses. For example, the *NAC* genes are transcriptionally regulated by the presence of stress-responsive regulatory elements such as DREs, ABREs, jasmonic acid responsive element, salicylic acid responsive element, LTREs, MYB and MYC (Myelocytomatosis) binding sites [23]. The W box regulates the expression of defense-related (*PR10*) genes and has roles in biotic and abiotic stresses, seed dormancy, and senescence [24, 25]. Overexpression of TaWRKY2 enhanced drought tolerance in wheat [26]. The gene *SNAC2* (stress responsive NAC gene) and ONAC022 (NAC gene) were found to be associated with cold, salt and osmotic tolerance in rice [27, 28]. Similarly, enhanced drought and salt tolerance was observed in transgenic cotton, tomato and *Arabidopsis* [29–31]. Consistent with the putative function in stress tolerance, qRT-PCR was performed to further check whether the expression of *TaOAT*s was induced by stress conditions. We found that *TaOAT* expression was markedly induced upon PEG and NaCl stress treatments and interestingly, its expression was highly induced in the drought tolerant cultivars as compared to the susceptible cultivars. Previous reports have shown that overexpression of *AtOAT* and *OsOAT* enhanced the oxidative stress tolerance in rice [13, 21]. Here, our data also indicated that TaOATs are also involved in wheat response to drought and salinity.

The presence of the plant AP-2-like *cis*-acting element directed our attention toward its role in floret development. Studies have shown that the AP-2-like domain was involved in the determination of meristematic cell fate during spikelet development, which results in different spikelet types such as branched silkless1/frizzy panicle1/branched floretless1 [32, 33]. Moreover, AP2 transcripts are the targets of microRNA172 (miR172), which help in the layer formation during spike and floret development [34–36]. To dissect this function of *OAT* genes in wheat, qRT-PCR was performed to determine the expression levels of *TaOAT* in different tissues (leaf, root, stem, seed, glume, lema, palea, stamens and pistil). To further investigate expression levels of *TaOAT* genes, we examined expression at different developmental stages (tillering, booting, heading, and grain filling). The high expression of *TaOAT* observed in stamens and at the heading stage supports its role in floret development. During floret development, the higher expression of *TaOAT* at tipping (TP), heading (HD) and antheis (AN) stages further supports the role of *TaOAT* in floret development. A recently study on rice reported that *OsOAT* mutant lines had a role in floret development. Moreover, the *OsOAT* defects of mutant rice affected the pollination process, causing low seed-setting rates and deformed seed shape [8]. Phylogenetic analysis demonstrated that OAT is highly conserved among cereals and exhibited similar gene structure and function. Our results together with recent reports on rice strongly support that *TaOATs* have a role in the pollination process via anther dehiscence based on the abnormalities observed in the *OsOAT* mutant.

Protein-protein interaction analysis showed that *TaOAT-5BL* interacts with proline biosynthesis genes (*TaP5CS* and *TaP5CR*) and nitrogen utilization gene (*TaARG*). Involvement of *OAT* gene in proline biosynthesis and nitrogen metabolism had also been confirmed in previous studies [6–11]. The crosslink of proline, arginine, and ornithine pathways have been also reviewed previously and it is found that ornithine (the precursor of OAT enzyme) occupies an important position in the three pathways [18]. Furthermore, wheat arginase gene has been reported to participate in nitrogen metabolism [16]. In this study, a positive interaction of *TaARG* and *TaOAT* was observed by Y2H assay. Together with the findings of this study, we deduced that *TaOATs* have significant roles in proline biosynthesis and are likely involved in arginine metabolism. Thus, we inferred that *TaOAT* has a potential role in nitrogen metabolism.

Plant OAT is thought to be involved in stress induced proline biosynthesis. Several studies demonstrated the positive contribution of OAT in proline synthesis during abiotic stress condition. It was found that *AtOAT* gene played a positive role for salt tolerance in young *Arabidopsis* seedlings and participated in proline biosynthesis [11]. A transgenic study demonstrated that the overexpression of *AtOAT* provides additional pathway for proline biosynthesis in *Nicotiana plumbaginifolia* via increasing OAT activity [22]. Similarly, overexpression of *AtOAT* in rice increases the proline content by 5 to 15-fold compared with that in wild type plants under salt and drought stress conditions [21]. Recently, it was demonstrated that the overexpression of *OsOAT* enhanced osmotic tolerance in transgenic rice [13]. Our present study showed that the overexpression of *TaOAT-5BL* in wheat significantly enhanced proline content in the transgenic lines under drought stress condition. Based on our results and previous studies, it can be concluded that *TaOAT* enhanced drought tolerances in transgenic plants via modifying proline biosynthesis. The transgenic wheat plants also showed an enhanced tolerance to in vitro salt stress condition. From these results we can summarize that *TaOAT* plays a significant role in drought and salt tolerance in wheat.

## Conclusion

In this study three copies of *TaOAT* genes, *TaAOT-5AL*, *TaOAT-5BL* and *TaAOT-5DL*, were cloned and localized on the long arm of chromosome 5 in wheat. We determined that due to the phenomenon of alternative splicing, two types of transcripts exist for *TaOAT-5AL*. Similar to *OsOAT* and *AtOAT*, *TaOATs* also target to mitochondria. Phylogenetic analysis showed that this enzyme is highly conserved among monocots as well as among dicots species. In silico promoter analysis revealed quite a number of *cis*-acting elements in the promoter region of the *TaOAT* gene, suggesting its role in drought- and salinity-stresses. Furthermore, qRT-PCR analysis showed the upregulation of the *TaOAT* gene in response to PEG and salt stress which supports its potential role in response to both of these stresses. The transgenic wheat plants overexpressing *TaOAT* displayed an increased tolerances to drought and salt stress conditions. Additionally, the presence of the plant AP-2-like *cis*-acting element and high expression of *TaOAT* in stamens suggest its role in floret development. The highest expression of *TaOAT* at the heading stage in combination with high expression of *TaOAT* in stamens suggested that it plays an important role in anther dehiscence and glume opening.

## Methods

### Plant materials and vectors

Common wheat varieties/lines Fielder, Chinese Spring, Yangmai158, Xiaobaimai, Jinmai47, and Hanxuan10 were acquired from the National Crop Germplasm Bank at the Institute of Crop Sciences (ICS), Chinese Academy of Agricultural Sciences (CAAS), for gene expression analyses under drought and salt stresses in this study. Fielder, Chinese Spring, and Yangmai158 are sensitive to drought, and Xiaobaimai, Jinmai47, and Hanxuan10 are tolerant to drought. Wheat lines Fielder and Xiaobaimai were used for *TaOAT* gene cloning and protoplast isolation in the subcellular localization experiment, respectively. Fielder was also planted in a growth chamber with controlled condition for expression analysis of *TaOAT* gene in different tissues and *Agrobacterium*-mediated transformation. Chinese Spring nullitetrasomic lines N5A/T5B, N5B/T5A, and N5D/T5A used for chromosomal location were provided by Prof. Zhishan Lin at ICS, CAAS. Vector p16318 for the subcellular localization of the target genes was provided Prof. Zhaoshi Xu at ICS, CAAS. Bait vector *pGBKT7* and pray vector *pGADT7* for yeast two hybrid assay were purchased from Clontech Laboratories (Takara Bio USA, Inc.). Expression vector *pWMB110* for wheat transformation was constructed by our laboratory previously [37].

### Sequence retrieval

*Arabidopsis AtOAT* (accession# At5g46180) was used as a query sequence to perform BLASTn using the IWGSC database (https://urgi.versailles.inra.fr/) against the IWGSC Ref Seq v1.0 for all chromosome scaffolds using default parameters. Gene structure was predicted based on the online FGENESH+ tool (http://www.softberry.com/), which was further confirmed by the Ensembl Plants database (http://plants.ensembl.org/index.html) and illustrated using GSDS2.0 (Gene Structure Display Server 2.0, http://gsds.cbi.pku.edu.cn/index.php).

### Extraction of gDNA and total RNA and synthesis of cDNA

The gDNA was extracted using NuClean plant genomic DNA kit (CWbio Inc., Beijing China). The DNA pallet was dissolved in ddH_2_O and quantified for further use. Total RNA was extracted from different plant tissues (i.e. root, stem leaf, seed, glume, lemma, palea, stamen, and pistil) and different developmental stages (*i.e* tillering, booting, heading and grain filling). To further investigate *TaOAT* expression in floret development, spikes were collected at nine different spike-developmental periods. These spikelet development periods were divided into seven spikelet developmental stages according to wheat and barley scales [38, 39]. Total RNA was extracted using the TRIzol Kit (TianGen Biotech Beijing Co., Ltd) and cDNA was synthesized using the cDNA synthesis kit (CWbio Inc., Beijing China) according to the respective manufacturer’s protocols and kept at − 20 °C for further analysis.

### Cloning of full-length *TaOAT* genes

Sequence alignment was performed for putative *TaOAT* genes on DNAMAN (Lynnon Corporation). Genome-specific primer pairs (GSP) were designed based on putative 5′- and 3′-untranslated regions (UTR) using primer premier 6 (Premier Biosoft) (Additional file 1: Table S3). The PCR components included 50–100 ng cDNA, 0.2 mM of each dNTP, 2 μl of 10× KOD buffer, 1 mM MgSO_4_, 0.5 μM each of the forward and reverse primer, 1 U KOD (Toyobo), amd 0.6 ul of 10% DMSO to a total volume of 20 μl. The PCR reactions were run on an ABI thermal cycler (ProFlex PCR) using the following cycler conditions: initial denaturing temperature at 95 °C for 5 min, followed by 35 cycles of 95 °C for 30 s, 60 °C for 30 s, 72 °C for 1 min and 30 s with a final extension at 72 °C for 8 min. Isolation of *TaOAT-5DL* was performed by nested-PCR or two-step PCR under thermal cycler conditions of 98 °C for 4 min, followed by 35 cycles of 98 °C for 15 s, 68 °C for 1 min 30 s, and an 8-min final extension at 72 °C. The PCR products were ligated onto the* PLB* T simple vector (TIANGEN, China) and introduced into DH5α *E. coli* according to the manufacturer’s recommendations for sequencing.

### Chromosome localization

To confirm the chromosomal locations of the three alleles of wheat *TaOAT* genes, the Chinese Spring nullitetrasomic lines involved in wheat chromosome group 5 were employed and amplified by PCR using the GSP (Additional file 2: Table S3). The PCR conditions were the same as mentioned in the above description with the exception of an extension time of 50 s.

### Subcellular localization of TaOAT

Prediction of the subcellular localization of OATs was performed using TargetP (http://www.cbs.dtu.dk/services/TargetP/) and Mitoprot (https://ihg.gsf.de/ihg/mitoprot.html). The conserved sequence from *OAT* genes was illustrated by WebLogo (http://weblogo.threeplusone.com/). The names of species and their predictions are listed in the (Additional file 2: Table S1). To confirm the prediction, the full ORF of *TaOAT-5BL* was in-frame fused upstream of a green fluorescent protein (GFP) vector *p16318* under the control of a 35S promoter. For transient expression assays, wheat protoplasts were isolated from 14-day-old seedlings and transformed with 1 μg of plasmids. Transformed protoplasts were incubated in the dark at 28 °C for 16–20 h. After that, a mitochondrion-specific dye (MitoTracker Orange, Invitrogen) was used to stain mitochondria and then protoplasts were subjected to microscopic examination under 488- and 543-nm illumination using a Zeiss LSM700 microscope (ZEISS Germany).

### In silico analysis

A phylogenetic tree was constructed using Mega 6.0 software (http://www.megasoſtware.net). The maximum likelihood method was employed with 1000 bootstrap replicates. BLASTp search was carried out to retrieve sequences by using the TaOAT amino acid sequences as queries in the NCBI and IWGSC protein databases. In total, 65 species including 20 from monocotyledonous plant species and 45 from dicotyledonous plant species were used in the analysis and their names and accession numbers are given in (Additional file 2: Table S2). Redundant sequences from the same plant species were removed. To find the motifs involved in regulation of drought-response, the 1000 base pairs upstream of each gene (*TaOAT-5AL*, *TaOAT-5BL* and *TaOAT-5DL*) from the start codon were selected to investigate the *cis*-acting elements using the PlantCARE database (http://bioinformatics.psb.ugent.be/webtools/plantcare/html/). To explore putative functions of TaOAT, we used the STRING database (https://string-db.org/) of protein-protein interactions.

### Yeast two hybrid assay

The predicted STRING interaction between TaOAT and TaARG was tested by yeast two hybrid assay to validate the results. *TaOAT-5BL* encoding sequence without transmembrane helix was cloned into bait vector *pGBKT7* while the coding sequence of *TaARG-2BS* without transmembrane helix was cloned into pray vector *pGADT7* using specific primers (Additional file 2: Table S3). The bait and prey vectors were cotransformed into freshly prepared yeast competent cells according to manufacturing protocol (Yeastmaker™ Yeast Trasforation System Two, Takara Bio Clontch laborties, Inc.).

### Vector construction and wheat transformation

The allele *TaOAT-5BL* was slected as a target gene to construct overexpression vector. The target gene was digested with *BamH1* and *Smal* enzymes and then inserted onto the digested *pWMB110* (Additional file 1: Figure S9) with the same enzymes at the MCS under the control of maize ubi promoter to form a new recombination vector *pWMB206* (Fig. 8a). The new vector was transformed into *Agrobacterium tumefaciens* strain C58C1 by triparental mating [40] and further introduced into the immature embryos of wheat variety Fielder to generate transgenic plants using the methods described previously [37, 41].

### Detection of transgenic wheat plants

Leaf fragment samples were collected from putative transgenic wheat plants and detected by a QuickStix Kit (EnviroLogix, USA) for the selection marker of *bar* gene by the manufacturer’s instruction and our previous publication [41]. The presence of *TaOAT-5BL* was decteted by PCR amplification using its specific primer (Additional file 2: Table S3). The PCR reactions were run on an ABI thermal cycler (ProFlex PCR) using the following cycler conditions: initial denaturing temperature at 95 °C for 5 min, followed by 35 cycles of 95 °C for 30 s, 60 °C for 30 s, 72 °C for 1 min and 30 s with a final extension at 72 °C for 8 min. Transgenic plants were self-crossed for 2 times for momozygotes accompanying PCR test, and 3 independent stable transgenic lines were selected for functional analysis. The expression of *TaOAT-5BL* was checked by semi-quantative PCR using specific primer (Additional file 2: Table S3). The PCR reactions were run on an ABI thermal cycler (ProFlex PCR) using the following cycler conditions: initial denaturing temperature at 95 °C for 5 min, followed by 25 cycles of 95 °C for 30 s, 60 °C for 30 s, 72 °C for 20 s with a final extension at 72 °C for 5 min.

### Stress treatment designing

Mature seeds of wheat from three drought tolerant cultivars (Xiaobaimai, Jinmai47, and Hanxuan10) and three susceptible cultivars (Fielder, Chinese Spring, and Yangmai158) were surface sterilized with 70% ethanol and then washed two times with sterilized water. The seeds were germinated in petri dishes containing filter paper soaked with distilled water. One week after germination, the seedlings were transformed into bigger jars (6.5 mm in diameter) and kept moist. When the primary leaves reached approximately 20 cm in length, seedlings were exposed to a 50%-PEG4000 stress. The leaves were collected after 0, 5, 10, 20, 40 h of abiotic stress treatment and immediately frozen in liquid nitrogen for RNA extraction. Similarly, for the salinity-stress test, 200 mM NaCl solution was added to jars of plants at the seedling stage. The leaf samples were collected at 0, 10, 20, and 40 h after salt stress.

Ten mature embryos of the *TaOAT-5BL* overexpressed wheat lines and the wild type were inoculated after sterilization with 25% sodium hypocholoride and 70% alcohol, in a culture box with three replicates, containing 1/2 MS medium with 150 mmol NaCl to identify their salt tolerance. The boxes were then cultured at 25 °C for 30 d under light condition with a optical density of 100 μmol m^− 2^ s^− 1^ and a photoperiod of 16 h light/8 h dark. Eight days later after the inoculation, germination percentage was calculated. Thirty days later after the inoculation, survival rate and root and shoot length were measured. For drought tolerance test, transgenic plants and wild type were sown in a tray containing natural soil from field and maintained in a glasshouse with a 16 h day at 24 °C and a 8 h night at 16 °C. Water withholding was started at three-leaf-stage. Free proline level in the wheat plants was measured at17 days after water withholding according to the method previously described [42].

### Quantitative real-time RT-PCR

Based on the conserved domain in the *TaOAT* ORF region, gene-specific primer pairs were designed. The qRT-PCR was performed on the Applied Biosystems™ 7500 Real-Time PCR Systems. The total volume of the PCR reaction was 20 μl. Each reaction contained 10 μl 2× SYBR Premix Ex Taq, 400 ng of cDNA, 0.2 μM of each primer, 0.4 μl ROX Reference Dye II, and 6.8 μl ddH_2_O (SYBR PrimeScript RT-PCR Kit, TaKaRa, Dalian, China). The PCR samples were preheated at 95 °C for 10 min, followed by 40–45 cycles of 95 °C for 15 s, 60 °C for 30 s, and 72 °C for 30 s. A constitutively expressed wheat gene, *TaADP*, was used for normalization of gene transcript level. All reactions were conducted in triplicate from three biological replicates.

## Supplementary information

**Additional file 1 **: **Figure S1.** Gene structures of *TaOAT-5AL* from hexapolid wheat (**a**) and tetraploid wheat (**b**). **Figure S2.** Presence of T → C substitution that caused the two types of *TaOAT-5AL* transcript in comparison to the one type of *TaOAT-5BL* and *TaOAT-5DL* transcipts. **Figure S3.** Phylogenetic tree of plant OAT proteins. The maximum likelihood tree was constructed based on amino acid sequence alignment. The three letters following OAT are abbreviations for plant names in *Latin* (Additional file 2: Table S2). The numbers at the nodes indicate the level of confidence for the major branches determined by bootstrap analysis. **Figure S4.** WebLogo representation of 65 plant species shows conservation of more than 75% in sequence. **Figure S5***Cis*-acting elements found in the promoter region of *TaOAT* genes. **Figure S6** Predicted functional partners of *TaOAT* gene. All the colored nods represent the significant first shell of interaction with *TaOAT-5BL* gene. Neighborhood, gene fusion and co-occurrence are categorized into predicted interaction based on interaction frequently observed in other species genomes or gene family occurrence across the genome. Experiment shows the interaction determined by laboratory experiment carried in other species and information was transferred to wheat to find the expected *TaOAT-5BL* interacting partner genes. Database shows known metabolic pathways in related species and then expected interaction in target species. Scores represent level of significant interaction. **Figure S7** Detection of positive *TaOAT-5BL* T_0_ plants. (**a**) Screening of positive *TaOAT-5BL* plants by PCR amplification using specific primer (Additional file 2: Table S3). 1–35: putative transgenic plants; P: expression vector *pWMB206* as positive control; N: wild type Fielder as negative control; W: water (**b**) Screening of positive plants by a QuickStix Kit. Two bands indicate positive plants and single band indicates the negative plants. **Figure S8** Germination status of the mature embryos of *TaOAT-5BL* transgenic lines and wild type Fielder on 150 mM salt containing medium. (**a**) Germination status after 8 days since inoculation. (**b**) Graphical representation of germination percentage of 3 transgenic lines (OE-F7, OE-F8, and OE-F9) and their wild type (FC). Data is the average of 3 replications and the statistical analysis was performed using IBM-SPSS statistic 20. Small letters in bold represent significant differences between groups at α < 0.05 using Duncan’s multiple range tests. Bars indicate the standard error of the mean. **Figure S9** The parent vector *pWMB110* used to construct the expression vector containing *TaOAT-5BL* gene for transformation in whaet.

**Additional file 2 **: **Table S1** Predictions of mitochondrial targeting of plant OAT using TargetP. **Table S2** Accession numbers of OAT protein sequences used in the phylogenetic analysis. **Table S3** The primers designed and used in the study.

**Additional file 3.** Sequences of interacting proteins.

## Data Availability

The sequences of the cloned genes in the current study were deposited to the National Center for Biotechnology Information (NCBI) and can be accessed in nucleotide database (https://www.ncbi.nlm.nih.gov/nuccore/?term=) or all database (https://www.ncbi.nlm.nih.gov/search/) as accession numbers MK942062, MK942063, MK680533 and MK748213 for *TaOAT-5AL-1*, *TaOAT-5AL-2*, *TaOAT-5BL*, and *TaOAT-5DL*, respectively. All materials are available from the authors upon request.

## Abbreviations

ABRE: Abscisic acid responsive element
bZIP: Basic leucine zipper motif
DRE: Dehydration responsive elements
ERE: Ethylene responsive elements
GSA: Glutamyl-5-semialdehyde
GSP: Genome-specific primers
HSE: Heat shock elements
IWGSC: International Wheat Genome Sequencing Consortium
LTR: Low temperature responsive element
NAC: NAM, ATAF, CUC
MYB: Myeloblastosis
OAT: Ornithine aminotransferase
ORF: Open reading frame
P5CR: Pyrroline-5-carboxylate reductase
P5CS: Delta 1-Pyrroline-5-carboxylate synthetase
PCR: Polymerase chain reaction
qRT-PCR: Quantative reverse transcription PCR
PEG: Polyethylene glycol
Pro: Proline
