# Supplementary material for: Cloning and molecular characterization of Triticum aestivum ornithine amino transferase (TaOAT) encoding genes
Source: BMC Plant Biol. 2020 Apr 29;20:187. doi: 10.1186/s12870-020-02396-2 (PMC7189522; doi:10.1186/s12870-020-02396-2)
Supplement: Supplementary file 1 — Additional file 1 : Figure S1. Gene structures of TaOAT-5AL from hexapolid wheat (a) and tetraploid wheat (b). Figure S2. Presence of T → C substitution that caused the two types of TaOAT-5AL transcript in comparison to the one type of TaOAT-5BL and TaOAT-5DL transcipts. Figure S3. Phylogenetic tree of plant OAT proteins. The maximum likelihood tree was constructed based on amino acid sequence alignment. The three letters following OAT are abbreviations for plant names in Latin (Additional file 2: Table S2). The numbers at the nodes indicate the level of confidence for the major branches determined by bootstrap analysis. Figure S4. WebLogo representation of 65 plant species shows conservation of more than 75% in sequence. Figure S5Cis-acting elements found in the promoter region of TaOAT genes. Figure S6 Predicted functional partners of TaOAT gene. All the colored nods represent the significant first shell of interaction with TaOAT-5BL gene. Neighborhood, gene fusion and co-occurrence are categorized into predicted interaction based on interaction frequently observed in other species genomes or gene family occurrence across the genome. Experiment shows the interaction determined by laboratory experiment carried in other species and information was transferred to wheat to find the expected TaOAT-5BL interacting partner genes. Database shows known metabolic pathways in related species and then expected interaction in target species. Scores represent level of significant interaction. Figure S7 Detection of positive TaOAT-5BL T0 plants. (a) Screening of positive TaOAT-5BL plants by PCR amplification using specific primer (Additional file 2: Table S3). 1–35: putative transgenic plants; P: expression vector pWMB206 as positive control; N: wild type Fielder as negative control; W: water (b) Screening of positive plants by a QuickStix Kit. Two bands indicate positive plants and single band indicates the negative plants. Figure S8 Germination status of the mature embryos of [file 12870_2020_2396_MOESM1_ESM.docx]

**Supplementary figures:**


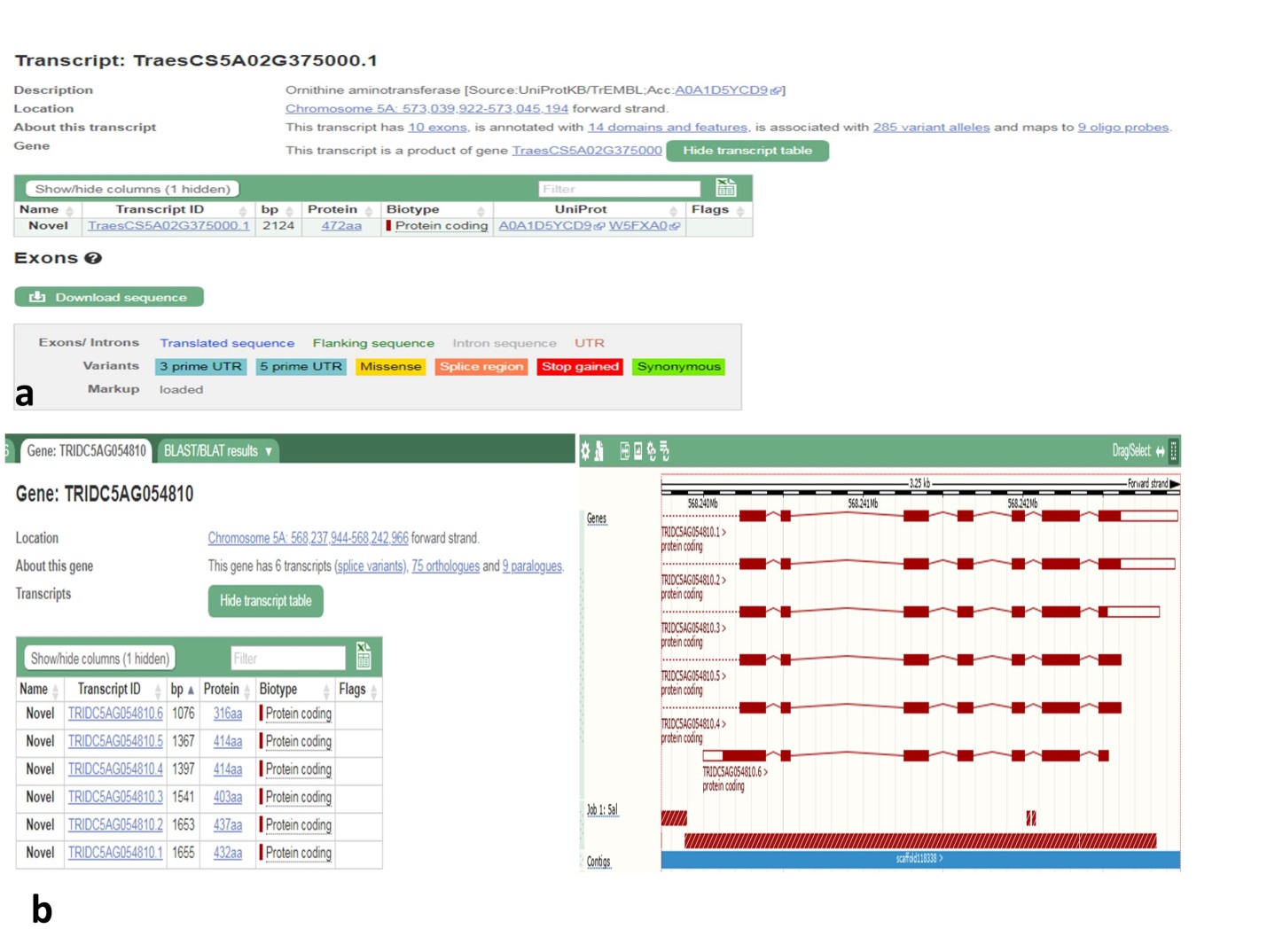


**Figure S1** Gene structures of *TaOAT-5AL* from hexapolid wheat (**a**) and tetraploid wheat (**b**).


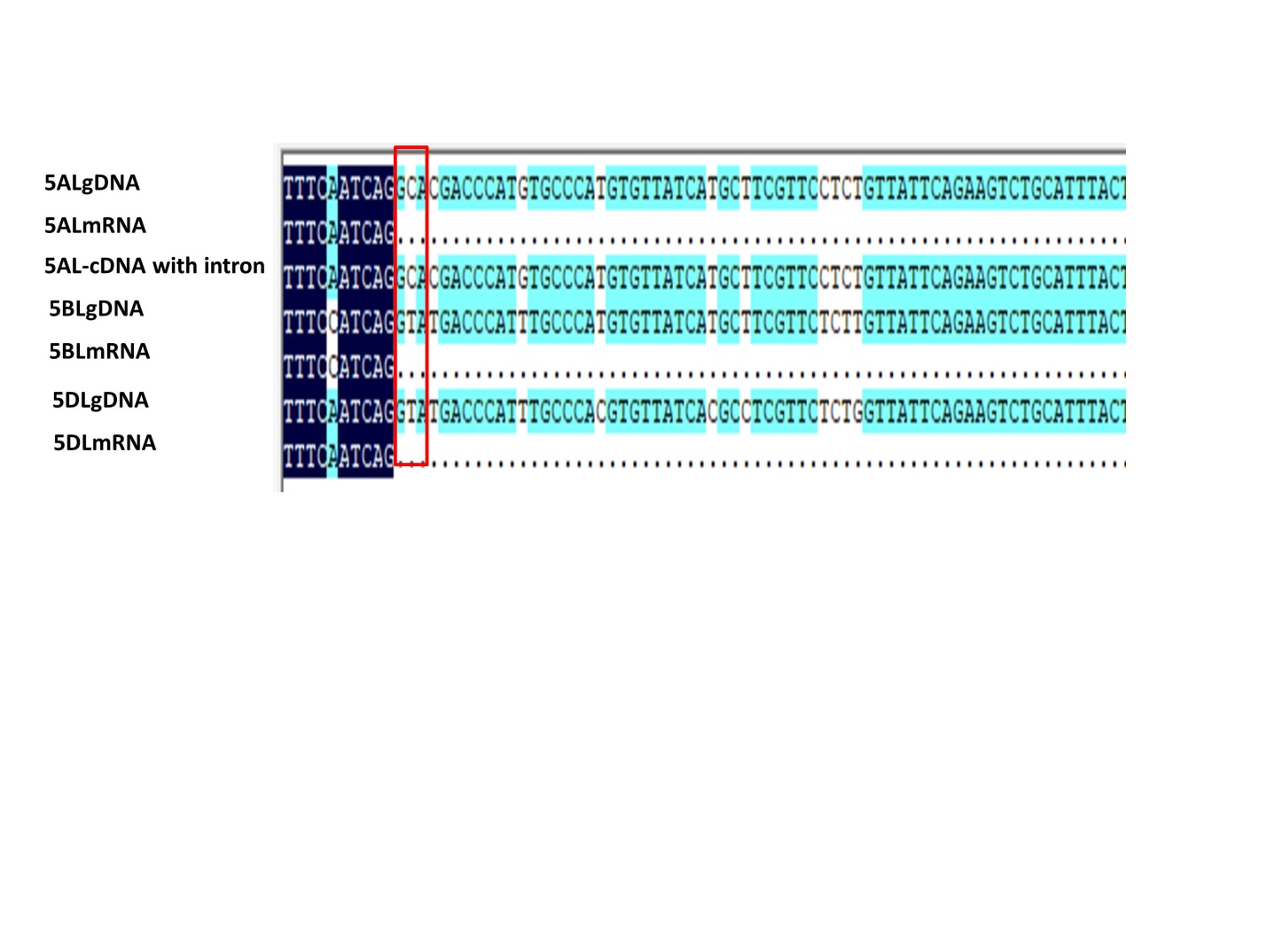


**Figure S2** Presence of T→C substitution that caused the two types of *TaOAT-5AL* transcript in comparison to the one type of *TaOAT-5BL* and *TaOAT-5DL* transcipts.


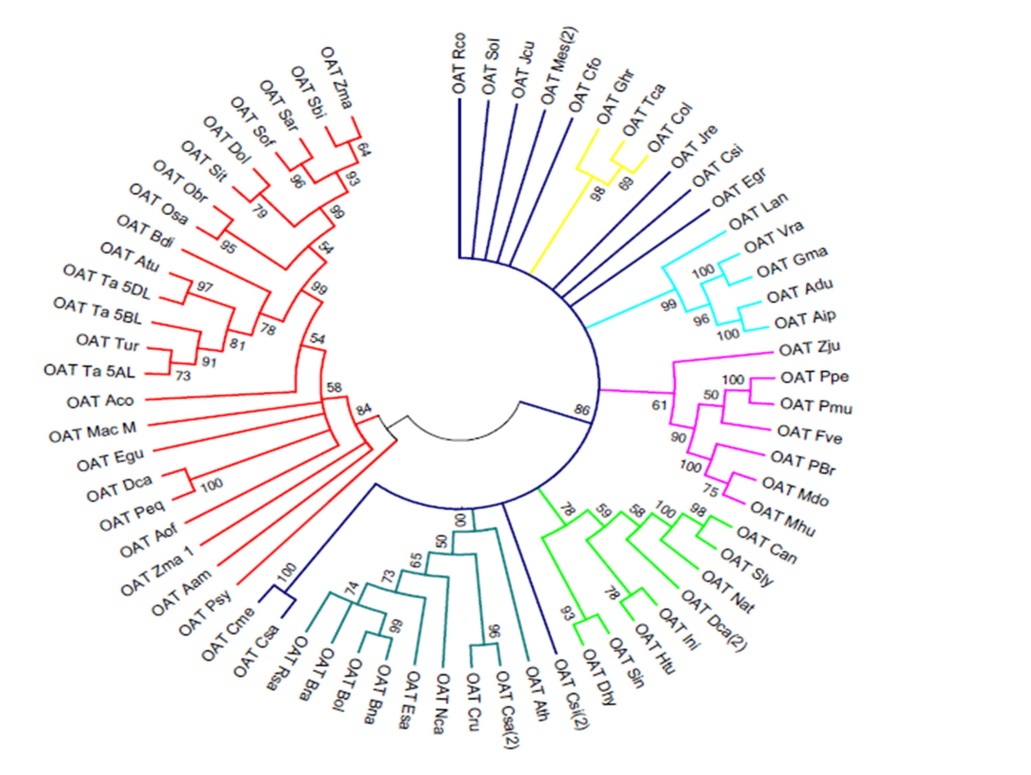


**Figure S3** Phylogenetic tree of plant OAT proteins. The maximum likelihood tree was constructed based on amino acid sequence alignment. The three letters following OAT are abbreviations for plant names in Latin (Additional file 2: Table S2). The numbers at the nodes indicate the level of confidence for the major branches determined by bootstrap analysis.

**
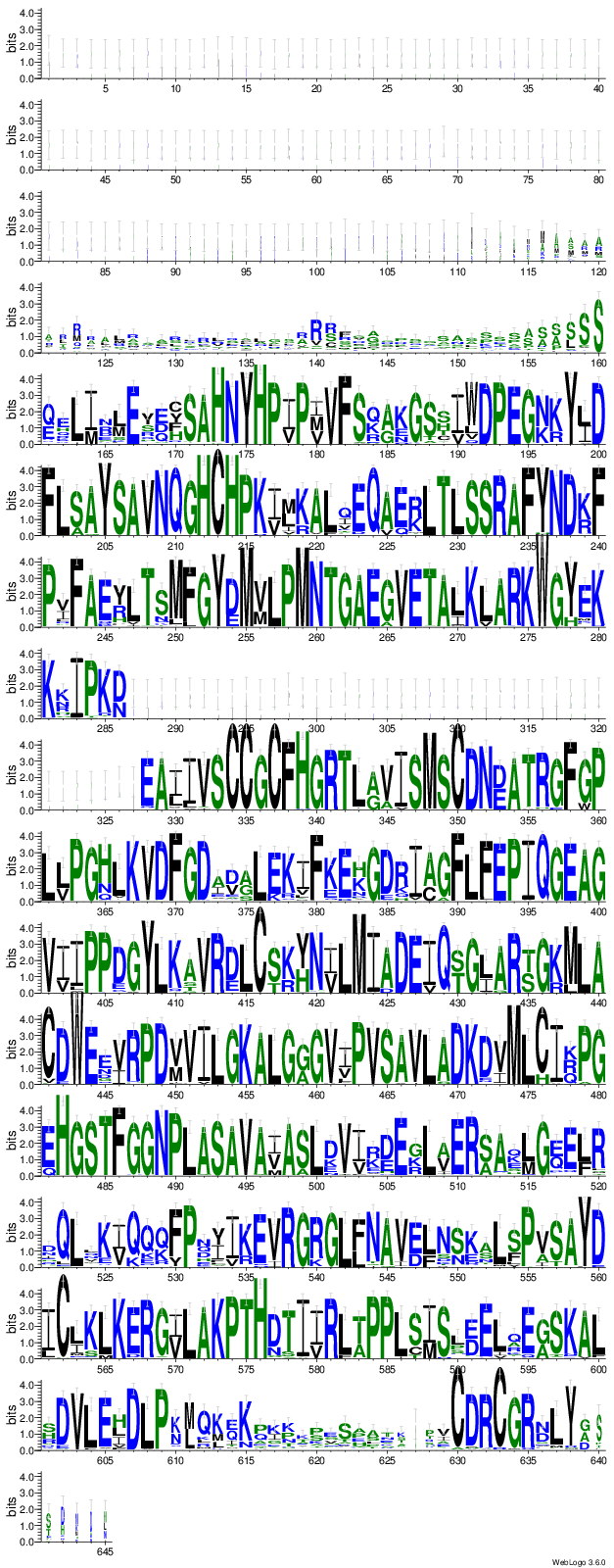
**

**Figure S4** WebLogo representation of 65 species shows conservation of more than 75% of sequence.


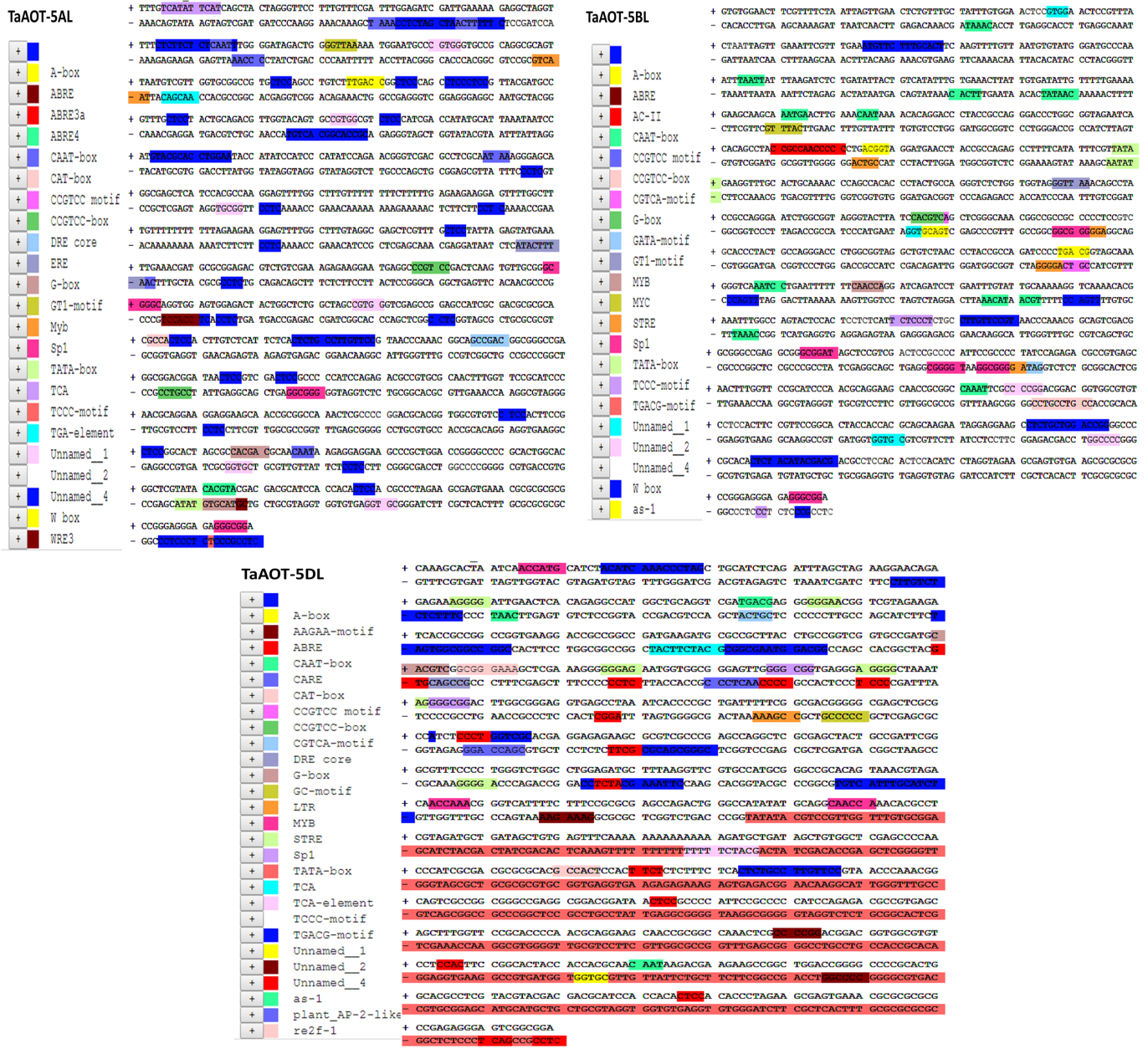


**Figure S5** *Cis*-acting elements found in the promoter region of *TaOAT* genes.


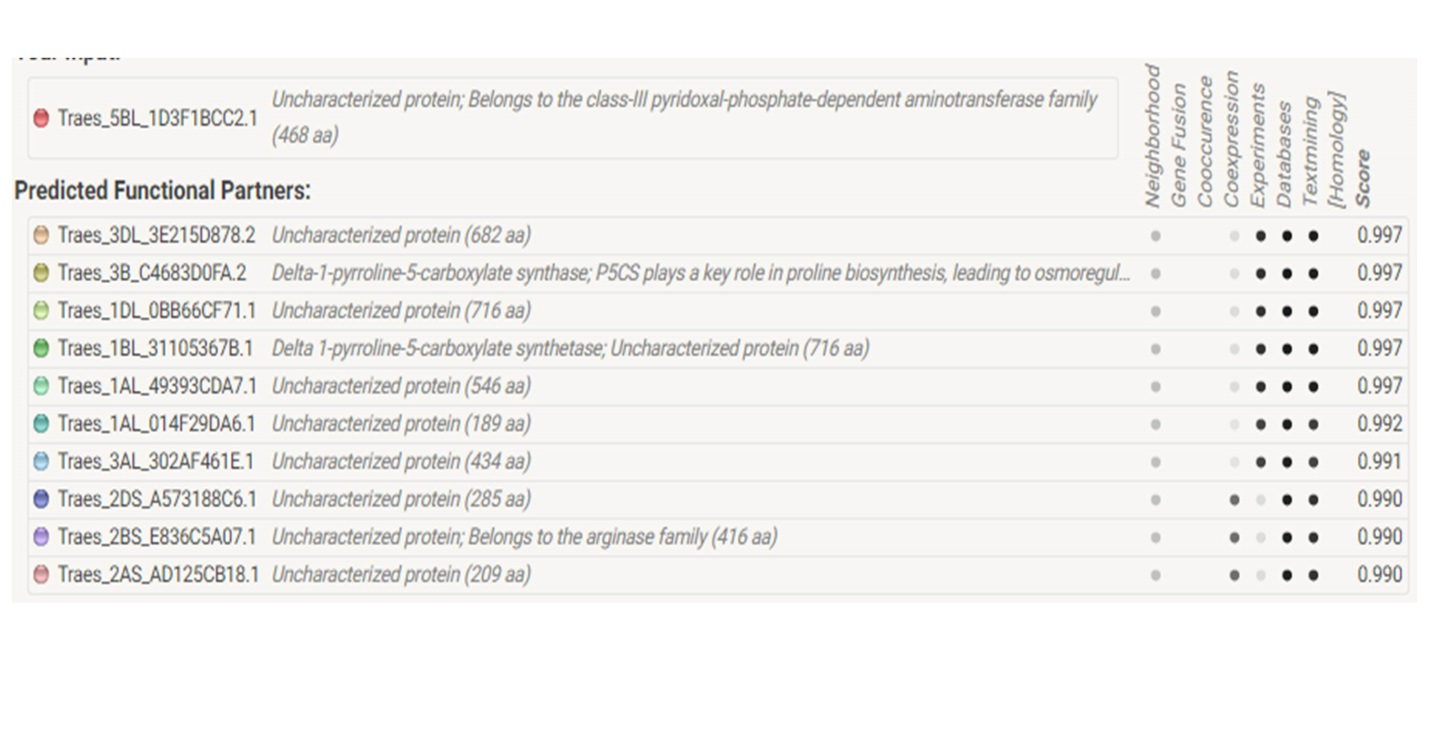


**Figure S6** Predicted functional partners of *TaOAT* gene. All the colored nods represent the significant first shell of interaction with *TaOAT-5BL* gene. Neighborhood, gene fusion and co-occurrence are categorized into predicted interaction based on interaction frequently observed in other species genomes or gene family occurrence across the genome. Experiment shows the interaction determined by laboratory experiment carried in other species and information was transferred to wheat to find the expected TaOAT-5BL interacting partner genes. Database shows known metabolic pathways in related species and then expected interaction in target species. Scores represent level of significant interaction.


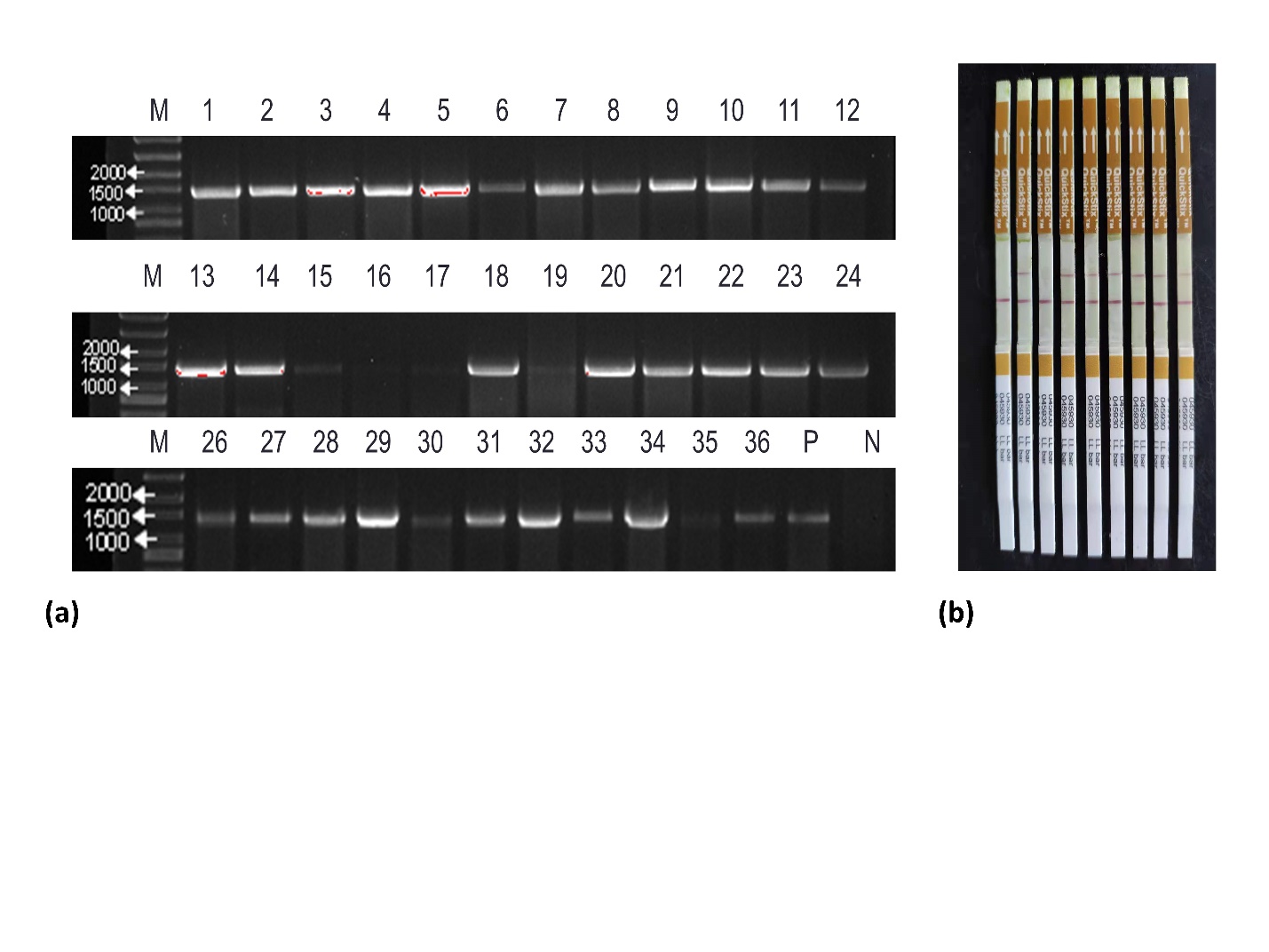


**Figure S7** Detection of positive *TaOAT-5BL* T_0_ plants. (**a**) Screening of positive *TaOAT-5BL* plants by PCR amplification using specific primer (Additional file 2: Table S3). 1-35: putative transgenic plants; P: expression vector *pWMB206* as positive control; N: wild type Fielder as negative control; W: water (**b**) Screening of positive plants by a QuickStix Kit. Two bands indicate positive plants and single band indicates the negative plants.


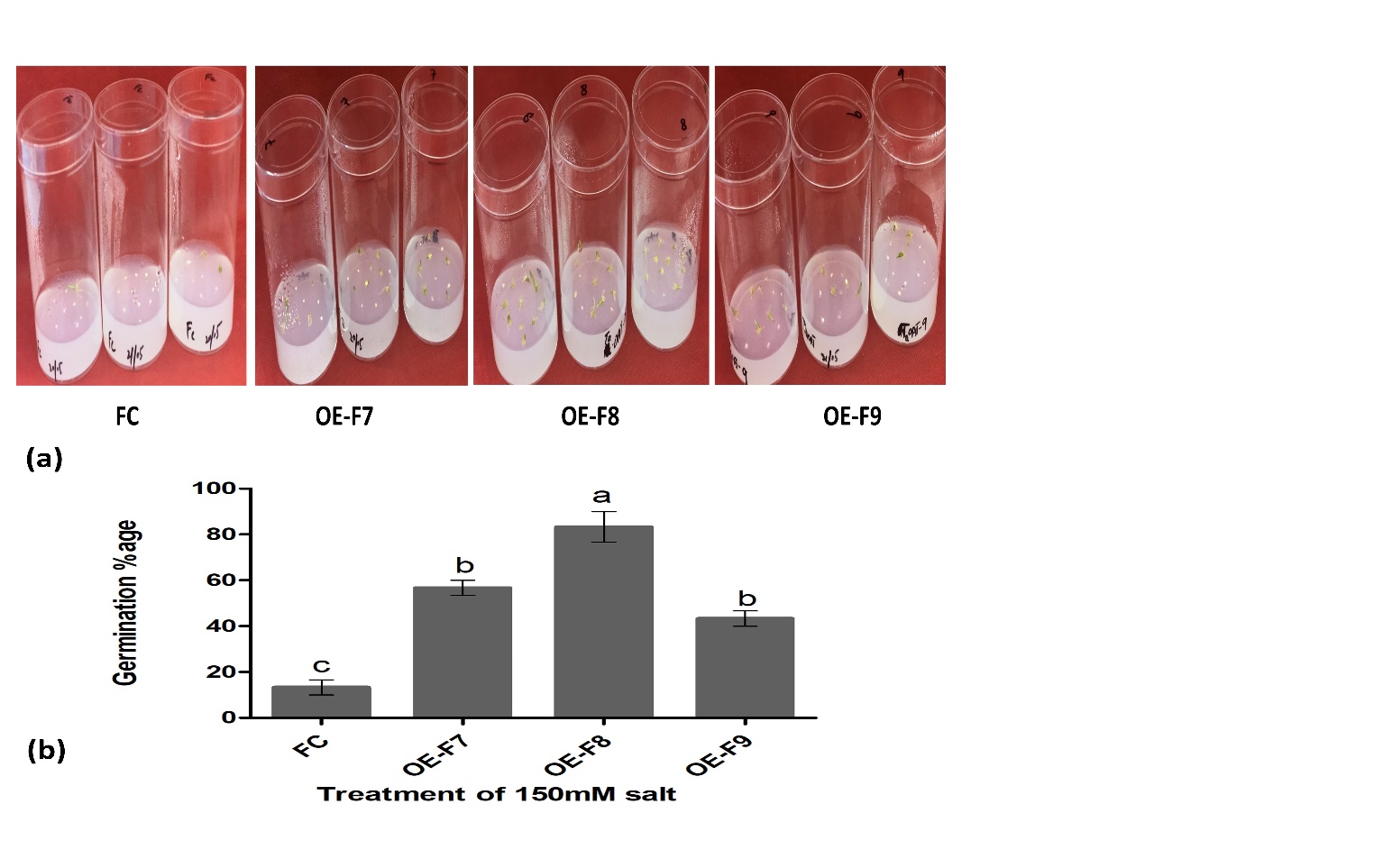


**Figure S8** Germination status of the mature embryos of *TaOAT-5BL* transgenic lines and wild type Fielder on 150 mM salt containing medium. (**a**) Germination status after 8 days since inoculation. (**b**) Graphical representation of germination percentage of 3 transgenic lines (OE-F7, OE-F8, and OE-F9) and their wild type (FC). Data is the average of 3 replications and the statistical analysis was performed using IBM-SPSS statistic 20. Small letters in bold represent significant differences between groups at α < 0.05 using Duncan’s multiple range tests. Bars indicate the standard error of the mean.


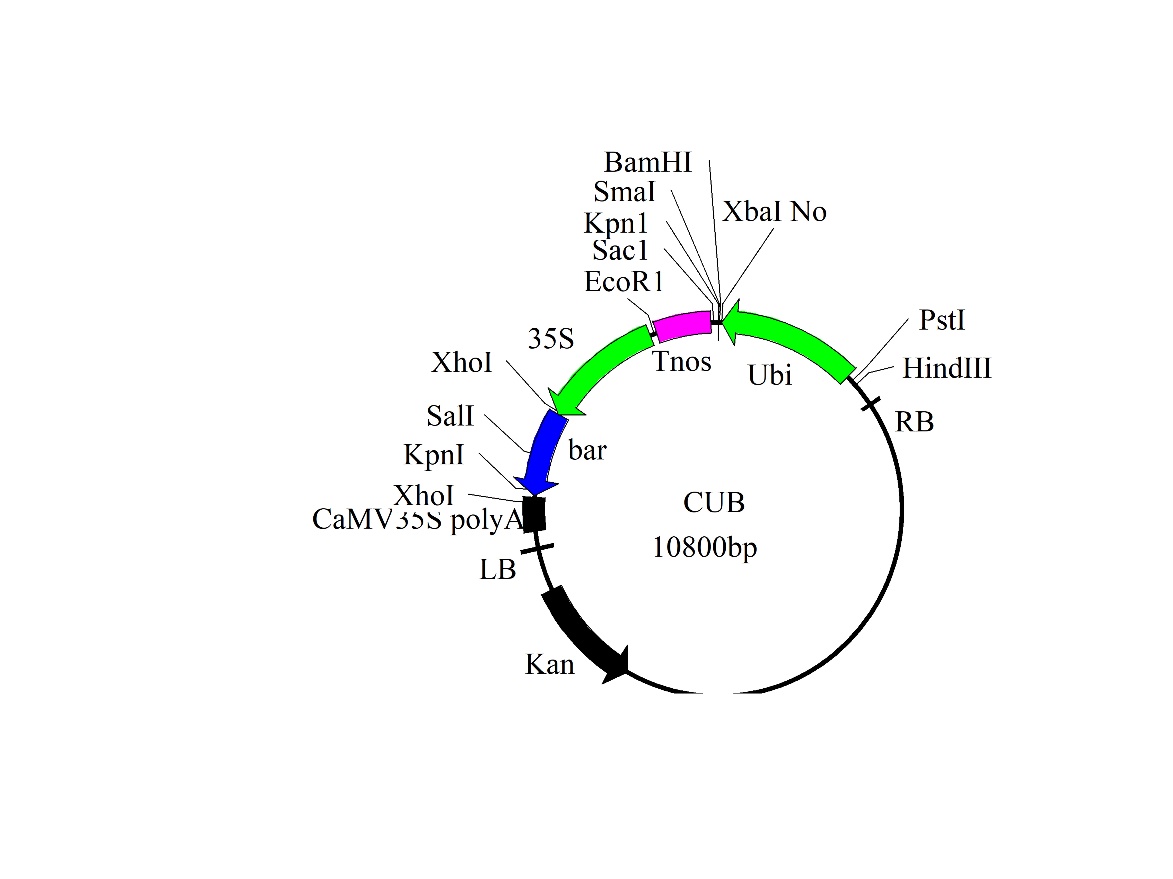


**Figure S9** The parent vector *pWMB110* used to construct the expression vector containing *TaOAT-5BL* gene for transformation in whaet.
