# Supplementary material for: Cloning and molecular characterization of Triticum aestivum ornithine amino transferase (TaOAT) encoding genes
Source: BMC Plant Biol. 2020 Apr 29;20:187. doi: 10.1186/s12870-020-02396-2 (PMC7189522; doi:10.1186/s12870-020-02396-2)
Supplement: Supplementary file 2 — Additional file 2 : Table S1 Predictions of mitochondrial targeting of plant OAT using TargetP. Table S2 Accession numbers of OAT protein sequences used in the phylogenetic analysis. Table S3 The primers designed and used in the study. [file 12870_2020_2396_MOESM2_ESM.docx]

**Table S1** Predictions of mitochondrial targeting of plant OAT using TargetP

| No. | Plant species | Length (aa) | cTP | mTP | SP | other | Localization* | RC |
| --- | --- | --- | --- | --- | --- | --- | --- | --- |
| 1 | *Triticum aestivum* | 468 | 0.079 | 0.9 | 0.109 | 0.008 | M | 2 |
| 2 | *Aegilops tauschii subsp. tauschii* | 473 | 0.156 | 0.865 | 0.055 | 0.007 | M | 2 |
| 3 | *Brachypodium distachyon* | 593 | 0.084 | 0.46 | 0.013 | 0.334 | M | 5 |
| 4 | *Triticum urartu* | 414 | 0.139 | 0.072 | 0.033 | 0.873 | _ | 2 |
| 5 | *Oryza sativa Indica Group* | 466 | 0.091 | 0.936 | 0.019 | 0.039 | M | 1 |
| 6 | *Saccharum officinarum* | 454 | 0.375 | 0.221 | 0.158 | 0.235 | C | 5 |
| 7 | *Saccharum arundinaceum* | 454 | 0.375 | 0.221 | 0.158 | 0.235 | C | 5 |
| 8 | *Sorghum bicolor* | 469 | 0.076 | 0.946 | 0.034 | 0.011 | M | 1 |
| 9 | *Setaria italica* (foxtail millet) | 477 | 0.095 | 0.811 | 0.138 | 0.003 | M | 2 |
| 10 | *Zea mays* | 472 | 0.193 | 0.946 | 0.024 | 0.009 | M | 2 |
| 11 | *oryza brachyantha* | 432 | 0.071 | 0.234 | 0.234 | 0.828 | _ | 3 |
| 12 | *Dichanthelium oligosanthes* | 448 | 0.299 | 0.238 | 0.198 | 0.196 | C | 5 |
| 13 | *Ananas comosus* | 475 | 0.14 | 0.898 | 0.027 | 0.024 | M | 2 |
| 14 | *Elaeis guineesis* | 474 | 0.213 | 0.852 | 0.034 | 0.039 | M | 2 |
| 15 | *Asparagus officinalis* | 484 | 0.811 | 0.599 | 0.003 | 0.01 | C | 4 |
| 16 | *Musa acuminata subsp. Malaccensis* | 477 | 0.12 | 0.935 | 0.014 | 0.026 | M | 1 |
| 17 | *Phalaenopsis equestris* | 470 | 0.191 | 0.752 | 0.024 | 0.081 | M | 3 |
| 18 | *Dendrobium catenatum* | 469 | 0.302 | 0.647 | 0.043 | 0.106 | M | 4 |
| 19 | *Anthurium amnicola* | 482 | 0.314 | 0.671 | 0.017 | 0.03 | M | 4 |
| 20 | *Prunus persica* | 472 | 0.377 | 0.675 | 0.035 | 0.019 | M | 4 |
| 21 | *Vigna radiata* | 399 | 0.054 | 0.379 | 0.057 | 0.688 | _ | 4 |
| 22 | *Prunus mume* | 472 | 0.234 | 0.721 | 0.053 | 0.013 | M | 3 |
| 23 | *Dendrobium catenatum* | 478 | 0.62 | 0.392 | 0.004 | 0.031 | C | 4 |
| 24 | *Fragaria vesca* | 484 | 0.587 | 0.674 | 0.007 | 0.006 | M | 5 |
| 25 | *Pyrus x bretschneideri* | 478 | 0.342 | 0.895 | 0.01 | 0.018 | M | 3 |
| 26 | *Malus domestica* | 478 | 0.358 | 0.881 | 0.01 | 0.02 | M | 3 |
| 27 | *Malus hupehensis* | 478 | 0.335 | 0.888 | 0.01 | 0.021 | M | 3 |
| 28 | *Ricinus communis* | 492 | 0.083 | 0.827 | 0.01 | 0.208 | M | 2 |
| 29 | *Cephalotus follicularis* | 468 | 0.043 | 0.86 | 0.027 | 0.089 | M | 2 |
| 30 | *Ipomoea nil* | 467 | 0.44 | 0.841 | 0.013 | 0.012 | M | 3 |
| 31 | *Jatropha curcas* | 474 | 0.178 | 0.567 | 0.047 | 0.109 | M | 4 |
| 32 | *Cucumis sativus* | 473 | 0.11 | 0.887 | 0.042 | 0.034 | M | 2 |
| 33 | *Juglans regia* | 474 | 0.134 | 0.732 | 0.047 | 0.094 | M | 3 |
| 34 | *Zostera marina* | 475 | 0.302 | 0.296 | 0.069 | 0.072 | C | 5 |
| 35 | *Cucumis melo* | 473 | 0.17 | 0.823 | 0.051 | 0.035 | M | 2 |
| 36 | *Camellia sinensis* | 473 | 0.163 | 0.758 | 0.022 | 0.115 | M | 3 |
| 37 | *Helianthus tuberosus* | 469 | 0.036 | 0.84 | 0.07 | 0.085 | M | 2 |
| 38 | *Nicotiana attenuata* | 474 | 0.048 | 0.885 | 0.026 | 0.082 | M | 1 |
| 39 | *Brassica napus* | 476 | 0.594 | 0.799 | 0.006 | 0.008 | M | 4 |
| 40 | *Brassica oleracea* | 476 | 0.594 | 0.799 | 0.006 | 0.008 | M | 4 |
| 41 | *Theobroma cacao* | 478 | 0.313 | 0.808 | 0.017 | 0.024 | M | 3 |
| 42 | *Brassica rapa* | 477 | 0.573 | 0.805 | 0.004 | 0.009 | M | 4 |
| 43 | *Capsicum annuum* | 463 | 0.205 | 0.584 | 0.061 | 0.045 | M | 4 |
| 44 | *Arachis duranensis* | 472 | 0.047 | 0.934 | 0.027 | 0.053 | M | 1 |
| 45 | *Arachis ipaensis* | 472 | 0.047 | 0.934 | 0.027 | 0.053 | M | 1 |
| 46 | *Solanum lycopersicum* | 469 | 0.328 | 0.83 | 0.015 | 0.04 | M | 3 |
| No. | Plant species | Length (aa) | cTP | mTP | SP | other | Localization* | RC |
| 47 | *Sesamum indicum* | 474 | 0.054 | 0.891 | 0.013 | 0.098 | M | 2 |
| 48 | *Spinacia oleracea* | 470 | 0.03 | 0.751 | 0.025 | 0.316 | M | 3 |
| 49 | *Noccaea caerulescens* | 473 | 0.448 | 0.89 | 0.011 | 0.011 | M | 3 |
| 50 | *Raphanus sativus* | 476 | 0.329 | 0.822 | 0.008 | 0.022 | M | 3 |
| 51 | *Eutrema salsugineum* | 504 | 0.797 | 0.11 | 0.004 | 0.31 | C | 3 |
| 52 | *Arabidopsis thaliana* | 475 | 0.48 | 0.88 | 0.004 | 0.01 | M | 3 |
| 53 | *Camelina sativa* | 477 | 0.256 | 0.908 | 0.003 | 0.015 | M | 2 |
| 54 | *Lupinus angustifolius* | 469 | 0.187 | 0.661 | 0.032 | 0.081 | M | 3 |
| 55 | *Citrus sinensis* | 470 | 0.085 | 0.877 | 0.016 | 0.07 | M | 2 |
| 56 | *Capsella rubella* | 475 | 0.657 | 0.81 | 0.004 | 0.009 | M | 5 |
| 57 | *Pinus sylvestris* | 468 | 0.036 | 0.933 | 0.019 | 0.108 | M | 1 |
| 58 | *Glycine max* | 467 | 0.059 | 0.842 | 0.029 | 0.137 | M | 2 |
| 59 | *Corchorus olitorius* | 469 | 0.152 | 0.092 | 0.096 | 0.754 | _ | 2 |
| 60 | *Dorcoceras hygrometricum* | 476 | 0.091 | 0.813 | 0.035 | 0.056 | M | 2 |
| 61 | *Ziziphus jujuba* | 485 | 0.192 | 0.569 | 0.021 | 0.14 | M | 4 |
| 62 | *Eucalyptus grandis* | 468 | 0.057 | 0.948 | 0.016 | 0.054 | M | 1 |
| 63 | *Malus domestica* | 478 | 0.358 | 0.881 | 0.01 | 0.02 | M | 3 |
| 64 | *Gossypium hirsutum* | 478 | 0.272 | 0.854 | 0.014 | 0.028 | M | 3 |
| 65 | *Manihot esculenta* | 473 | 0.092 | 0.888 | 0.033 | 0.045 | M | 2 |

cTP: a chloroplast transit peptide; mTP: a mitochondrial targeting peptide; SP: a signal peptide; RC: Reliability class, from 1 to 5, where 1 indicates the strongest prediction. Thus the lower the value of RC the safer the prediction. C, M, S, and _ mean cTP, mTP, SP, and any other location, respectively.

**Table S2** Accession numbers of OAT protein sequences used in the phylogenetic analysis

| No. | Plant species | Accession number | Annotation | No. of amino acid | Family |
| --- | --- | --- | --- | --- | --- |
| 1 | *Triticum aestivem-5AL* | MK942063 | OAT_Ta_5AL | 472 | monocot |
|  | *Triticum aestivem-5BL* | MK680533 | OAT_Ta_5BL | 468 | monocot |
|  | *Triticum aestivem-5DL* | MK748213 | OAT_Ta_5DL | 73 | monocot |
| 2 | *Aegilops tauschii subsp. tauschii* | XP_020174568.1 | OAT_Atu | 473 | monocot |
| 3 | *Brachypodium distachyon* | KQK13994.1 | OAT_Bdi | 593 | monocot |
| 4 | *Oryza sativa Indica Group* | EEC75822.1 | OAT_Osa-i | 466 | monocot |
| 5 | *Triticum urartu* | EMS55823.1 | OAT_Tur | 414 | monocot |
| 6 | *Zea mays* | NP_001130350.1 | OAT_Zma | 472 | monocot |
| 7 | *Saccharum officinarum* | ABP38411.1 | OAT_Sof | 454 | monocot |
| 8 | *Saccharum arundinaceum* | ABV03818.1 | OAT_Sar | 454 | monocot |
| 9 | *Sorghum bicolor* | XP_002464174.1 | OAT_Sbi | 477 | monocot |
| 10 | *Setaria italica (foxtail millet)* | XP_004982330.1 | OAT_Sit | 477 | monocot |
| 11 | *oryza brachyantha* | XP_006650339.1 | OAT_Obr | 432 | monocot |
| 12 | *Dichanthelium oligosanthes* | OEL26296.1 | OAT_Dol | 448 | monocot |
| 13 | *Ananas comosus* | XP_020092479.1 | OAT_Aco | 475 | monocot |
| 14 | *Elaeis guineesis* | XP_010940988.1 | OAT_Egu | 474 | monocot |
| 15 | *Asparagus officinalis* | ONK67313.1 | OAT_Aof | 484 | monocot |
| 16 | *Musa acuminata* | XP_009381301.1 | OAT_Mac | 477 | monocot |
| 17 | *Phalaenopsis equestris* | XP_020598662 | OAT_Peq | 470 | monocot |
| 18 | *Dendrobium catenatum* | XP_020697081 | OAT_Dca | 469 | monocot |
| 19 | *Anthurium amnicola* | JAT58794.1 | OAT_Aam | 482 | monocot |
| 20 | *Zostera marina* | KMZ72036.1 | OAT_Zma_1 | 475 | monocot |
| 21 | *Prunus persica* | ALT55650.1 | OAT_Ppe | 472 | eudicot |
| 22 | *Vigna radiata* | XP_014505671.1 | OAT_Vra | 469 | eudicot |
| 23 | *Manihot esculenta* | OAY29173.1 | OAT_Mes | 473 | eudicot |
| 24 | *Prunus mume* | XP_008225506.1 | OAT_Pmu | 472 | eudicot |
| 25 | *Dendrobium catenatum* | XP_017231196.1 | OAT_Dca-2 | 469 | eudicot |
| 26 | *Fragaria vesca* | XP_004293519.1 | OAT_Fve | 484 | eudicot |
| 27 | *Pyrus x bretschneideri* | XP_009360915.1 | OAT_PBr | 478 | eudicot |
| 28 | *Malus domestica* | XP_008345940.1 | OAT_Mdo | 478 | eudicot |
| 29 | *Malus hupehensis* | AEO51063.1 | OAT_Mhu | 478 | eudicot |
| 30 | *Ricinus communis* | XP_002519647.2 | OAT_Rco | 492 | eudicot |
| 31 | *Cephalotus follicularis* | GAV75030.1 | OAT_Cfo | 468 | eudicot |
| 32 | *Ipomoea nil* | XP_019157218.1 | OAT_Ini | 467 | eudicot |
| 33 | *Jatropha curcas* | NP_001306851.1 | OAT_Jcu | 474 | eudicot |
| 34 | *Cucumis sativus* | XP_004137365.1 | OAT_Csa | 473 | eudicot |
| 35 | *Juglans regia* | XP_018847636.1 | OAT_Jre | 474 | eudicot |
| 36 | *Cucumis melo* | XP_008444491.1 | OAT_Cme | 473 | eudicot |
| 37 | *Camellia sinensis* | AIC77166.1 | OAT_Csi | 473 | eudicot |
| 38 | *Helianthus tuberosus* | AHJ08571.1 | OAT_Htu | 469 | eudicot |
| 39 | *Nicotiana attenuata* | XP_019259981.1 | OAT_Nat | 474 | eudicot |
| 40 | *Brassica napus* | NP_001303219.1 | OAT_Bna | 476 | eudicot |
| 41 | *Brassica oleracea* | XP_013593040.1 | OAT_Bol | 476 | eudicot |
| 42 | *Theobroma cacao* | XP_017979303.1 | OAT_Tca | 478 | eudicot |
| 43 | *Brassica rapa* | NP_001288848.1 | OAT_Bra | 477 | eudicot |
| 44 | *Capsicum annuum* | XP_016537501.1 | OAT_Can | 463 | eudicot |
| 45 | *Arachis duranensis* | XP_015957870.1 | OAT_Adu | 472 | eudicot |
| 46 | *Arachis ipaensis* | XP_016191172.1 | OAT_Aip | 472 | eudicot |
| 47 | *Solanum lycopersicum* | XP_004244969.1 | OAT_Sly | 469 | eudicot |
| 48 | *Sesamum indicum* | XP_011096597.1 | OAT_Sin | 474 | eudicot |
| 49 | *Spinacia oleracea* | KNA17048.1 | OAT_Sol | 470 | eudicot |
| 50 | *Noccaea caerulescens* | JAU67277.1 | OAT_Nca | 473 | eudicot |
| 51 | *Raphanus sativus* | XP_018482908.1 | OAT_Rsa | 476 | eudicot |
| 52 | *Eutrema salsugineum* | XP_006398303.1 | OAT_Esa | 504 | eudicot |
| 53 | *Arabidopsis thaliana* | OAO92185.1 | OAT_Ath | 475 | eudicot |
| 54 | *Camelina sativa* | XP_010494787.1 | OAT_Csa-2 | 477 | eudicot |
| 55 | *Lupinus angustifolius* | XP_019419862.1 | OAT_Lan | 469 | eudicot |
| 56 | *Citrus sinensis* | XP_006468034.1 | OAT_Csi-2 | 470 | eudicot |
| 57 | *Capsella rubella* | XP_006280404.1 | OAT_Cru | 475 | eudicot |
| 58 | *Pinus sylvestris* | CAJ76070.1 | OAT_Psy | 468 | eudicot |
| 59 | *Glycine max* | XP_003531161.1 | OAT_Gma | 467 | eudicot |
| 60 | *Corchorus olitorius* | OMO65722.1 | OAT_Col | 469 | eudicot |
| 61 | *Dorcoceras hygrometricum* | KZV39047.1 | OAT_Dhy | 476 | eudicot |
| 62 | *Ziziphus jujuba* | XP_015875969.1 | OAT_Zju | 485 | eudicot |
| 63 | *Eucalyptus grandis* | XP_010052050.1 | OAT_Egr | 468 | eudicot |
| 64 | *Gossypium hirsutum* | XP_016753478.1 | OAT_Ghr | 478 | eudicot |
| 65 | *Manihot esculenta* | OAY29173.1 | OAT_Mes | 473 | eudicot |

**Table S3** The primers designed and used in the study

| Gene name | Primer (5′→3′) | Annotation |
| --- | --- | --- |
| *TaOAT-5AL-F* | CACTGGCACGGCTCGTATAC | Specific to *TaOAT-5AL* |
| *TaOAT-5AL-R* | GAGTGCGACATGGTGCGGTAT |  |
| *TaOAT-5BL-F* | GCGGAGATGGCGGCGATAAT | Specific to *TaOAT-5BL* |
| *TaOAT-5BL-R* | GCTCTGCTTGGATGCACCCT |  |
| *TaOAT-5DL-1st-F* | AAGGATCCGCATCCACCACACTCCACAC | Specific to *TaOAT-5DL* |
| *TaOAT-5DL-1st-R* | GTACCTCATCTCTGCCCGGATGAACGG |  |
| *TaOAT-5DL-2nd-F* | AGAGGGAGTCGGCGGAGATG |  |
| *TaOAT-5DL-2nd-R* | GGATGAACGGTCGCCCTTTA |  |
| *5AL-GS-F* | CCTCACAGATCGGAATTGGTC | 5AL chromosome location |
| *5AL-GS-R* | CGCACGTTTTGAAGGGATC |  |
| *5BL-GS-F* | ACTTGCTTTAATACCTCGGATCG | 5BL chromosome location |
| *5BL-GSR* | CATCATCCGGGACAGTGTTAT |  |
| *5DL-GS-F* | AGCAATGCGATTTAATTACGG | 5DL chromosome location |
| *5DL-GS-R* | CGACATCATGTTGAGATTTGTAA |  |
| *5AL-1-F-check* | CTCCCATTTCAATCAGGCAC | 5AL-1 confirmation |
| *5AL-1R-check* | CGTGGGATAAGAGGAGCAATG |  |
| *TaOAT-5BL-GFP-F* | TATCTCTAGAGGATCCATGGCGGCGATAATATCGCGG | Ta5BL- p16318 |
| *TaOAT-5BL-GFP-R* | TGCTCACCATGGATCCTAAATCCCGACCGCACCTATCACAG |  |
| *TaOAT-5BL-F1* | GGATCCATGGCGGCGATAATATCGCGG | Construction of expression vector, screeing for positive transgenic plants. |
| *TaOAT-5BL-R1* | CCCGGGTTATAAATCCCGACCGCACCTATCACAG |  |
| *TaOAT-5BL-BD-F* | CATGGAGGCCGAATTCATGGCGGCGATAATATCGCGG | Cloning of bait vector *pGBKT7* |
| *TaOAT-5BL-BD-R* | GCAGGTCGACGGATCCTTAACTTCCATGTTCTCCCGGCTT |  |
| *TaARG-2BS-AD-F* | GGAGGCCAGT GAATTC ATGGGCGGCGCGGCGGCGGC | Cloning of bait vector *pGADT7* |
| *TaARG-2BS-AD-F* | CCGTATCGATGCCCA CCCGGG TCACCCGTCGACCGTGTCGCGC |  |
| *TaOAT-F* | AATGAACACTGGAGCCGAAGG | qRT-PCR |
| *TaOAT-R* | GACCAGGAACCAAAGGACCAA |  |
| *TaOAT-5BL-qRTF* | GATGTGCTCCGCCCCGGCA | 5BL-qRT |
| *TaOAT-5BL-qRTR* | CAATATATGCGATCCTTCCCCTTTGGAG |  |
| *ADP-F* | GCTCTCCAACAACATTGCCAAC | Endogenous control |
| *ADP-R* | GCTTCTGCCTGTCACATACGC |  |
