# Supplementary material for: Cloning and molecular characterization of Triticum aestivum ornithine amino transferase (TaOAT) encoding genes
Source: BMC Plant Biol. 2020 Apr 29;20:187. doi: 10.1186/s12870-020-02396-2 (PMC7189522; doi:10.1186/s12870-020-02396-2)
Supplement: Supplementary file 3 — Additional file 3. Sequences of interacting proteins. [file 12870_2020_2396_MOESM3_ESM.docx]

Sequences of interacting proteins

>Traes_1AL_014F29DA6.1 4565.Traes_1AL_014F29DA6.1 Uncharacterized protein

MYSHQSPFSLPQKIYLLILETLLVHKDLMKIPELNDILVALKTAGVNLYCGPVAHKVLGYPKADSLHLEYSSMACTVEIVDDVQSAIDHIHRYGSAHTDCVVTTDDKVAETFLRQVDSAAVLYNASTRFSDGARFGLGAEVGISTGRIHARGPVGVEGLLTTRWLLRGKGQVVNGDKDVEYTHKSLPLQ

>Traes_1AL_49393CDA7.1 4565.Traes_1AL_49393CDA7.1 Uncharacterized protein

MAGADPNRSFMKDVKRIIIKVGTAVITRNDGRLALGRIGALCEQVKDLNAQGYEVIMVTSGAVGVGRQRLRYRKLVNSSFADLQKPQMELDGKACAAVGQSGLMALYDMLFTQLDVSSSQLLVTDSDFDNSNFRERLRETVESLLELRVIPIFNENDAISTRKAPYEDSSGIFWDNDSLAGLLALELKADLLVLLSDVDGLYSGPPSEPSSKLIHTYIKEKHYHEITFGDKSRVGRGGMTAKVQAAVWASTGGVPVVITSGCASQSLVKVLRGEKIGTLFHKNASLWEPSKDTSVREMAVAARDCSRHLQNLSSEERKKILLDVADALEANEDLIRSENEADLAAAHEAGYESALVSRLTLKPGKIASLAKSVRTLANMEDPINEILKRTEVADGLVLEKTSCPLGVLLIIFESRPDALVQIASLAIRSGNGLLLKGGKEAMRSNAILHKVITNAIPDNVGEKLIGLITTRDEIADLLKHDDVIDLVIPRGSNKLVAQIKSSTKIPVLGHADGVCHVYIDKSADMDMAKRIVMDAKIDYPAACNAM

>Traes_1BL_31105367B.1 4565.Traes_1BL_31105367B.1 Delta 1-pyrroline-5-carboxylate synthetase; Uncharacterized protein

MAGADPNRSFMKDVKRIIIKVGTAVITRNDGRLALGRIGALCEQVKDLNAQGYEVIMVTSGAVGVGRQRLRYRKLVNSSFADLQKPQMELDGKACAAVGQSGLMALYDMLFTQLDVSSSQLLVTDSDFDNSNFRERLRETVESLLELRVIPIFNENDAISTRKAPYEDSSGIFWDNDSLAGLLALELKADLLVLLSDVDGLYSGPPSEPSSKLIHTYIKEKHYHEITFGDKSRVGRGGMTAKVQAAVWASTGGVPVVITSGCASQSLVKVLRGEKIGTLFHKNASLWEPSKETSVREMAVAARDCSRRLQNLSSEERKKILLDVADALEANEDLIRSENEADLAAAHEAGYESALVSRLTLKPGKIASLAKSVRTLANMEDPINEILKRTEVADGLVLEKTSCPLGVLLIIFESRPDALVQIASLAIRSGNGLLLKGGKEAMRSNAILHKVITNAIPDNVGEKLIGLITTRDEIADLLKHDDVIDLVIPRGSNKLVAQIKSSTKIPVLGHADGVCHVYIDKSADMDMAKRIVMDAKIDYPAACNAMETLLVHKDLMKTPELNDILVALKTAGVNLYCGPVAHKVLGYPKADSLHLEYSSMACTVEIVDDVQSAIDHIHRYGSAHTDCVVTTDDKVAETFLRQVDSAAVLYNASTRFSDGARFGLGAEVGISTGRIHARGPVGVEGLLTTRWLLRGKGQVVNGDKDVEYTHKSLPLQ

>Traes_1DL_0BB66CF71.1 4565.Traes_1DL_0BB66CF71.1 Uncharacterized protein

MAGADLNRSFIKDVKRIIIKVGTAVITRNDGRLALGRIGALCEQVKDLNAQGYEVIMVTSGAVGVGRQRLRYRKLVNSSFADLQKPQMELDGKACAAVGQSGLMALYDMLFTQLDVSSSQLLVTDSDFDNSNFRERLRETVESLLELRVIPIFNENDAISTRKAPYEDSSGIFWDNDSLAGLLALELKADLLVLLSDVDGLYSGPPSEPSSKLIHTYIKEKHYHEITFGDKSRVGRGGMTAKVQAAVWASTGGVPVVITSGCASQSLVKVLRGEKIGTLFHKNASLWEPSKDTSVREMAVAARDCSRRLQNLTSEERKKILVDVADALEANEDLIRSENEADLAAAHEAGYESALVSRLTLKPGKIASLAKSVRTLANMEDPINEILKRTEVADGLVLEKTSCPLGVLLIIFESRPDALVQIASLAIRSGNGLLLKGGKEAMRSNAILHKVITNAIPNNVGEKLIGLITTRDEIADLLKHDDVIDLVIPRGSNKLVAQIKSSTKIPVLGHADGVCHVYIDKSADMDMAKRIVMDAKIDYPAACNAMETLLVHKDLMKTPELNDILVALKTAGVNLYCGPVAHKVLGYPKADSLHLEYSSMACTVEIVDDVQSAIDHIHRYGSAHTDCVVTTDDKVAETFLRQVDSAAVLYNASTRFSDGARFGLGAEVGISTGRIHARGPVGVEGLLTTRWLLRGKGQVVNGDKDVEYTHKSLPLQ

>Traes_2AS_AD125CB18.1 4565.Traes_2AS_AD125CB18.1 Uncharacterized protein

QIDACNQRVCQNSDGRRSSSAVVLGGDHSISYPVVRAVSEKLGGPVDILHLDAHPDIYDCFEGNTYSHASSFARIMEGGYARRLLQVGLRSITKEGREQGKRFGVEQYEMRTFSRDREKLESLKLGEGVKGVYVSVDVDCLDPAFAPGVSHIEPGGLSFRDVLNILQNLQGDVVAGDVVEFNPQRDTVDGMTAMVAAKLVRELSAKISK

>Traes_2BS_E836C5A07.1 4565.Traes_2BS_E836C5A07.1 Uncharacterized protein; Belongs to the arginase family

MHLVYCAECFAISEHLKGHMNLLVEKQVSCTSLAWHFVTSLPSFLSSPQPLRSLAFSRRLRLLLQSSVPPLDDSSAMGGAAAATGAARWIQRLSAARISTEALERGQSRVIDASLTLIRERAKLKGELLRAMGGVKASATLLGVPLGHNSSFLQGPAFAPPRIREAIWCGSTNSSTEEGKELNDPRVLTDVGDVPIQEIRDCGVEDDRLMHVISESVKTVMDEDPLRPLVLGGDHSISYPVVRAVSEKLGGPVDILHLDAHPDIYDCFEGNTYSHASSFARIMEGGYARRLLQVGLRSITKEGREQGKRFGVEQYEMRTFSRDREKLENLKLGEGVKGVYVSVDVDCLDPAFAPGVSHIEPGGLSFRDVLNILQNLQGDVVAGDVVEFNPQRDTVDGMTAMVAAKLVRELSAKISK

>Traes_2DS_A573188C6.1 4565.Traes_2DS_A573188C6.1 Uncharacterized protein

MGGVKASATLLGVPLGHNSSFLQGPAFAPPRIREAIWCGSTNSSTEEGKELNDPRVLTDVGDVPIQEIRDCGVEDDRLMHVISESVKTVMDEDPLRPLVLGGDHSISYPVVRAVSEKLGGPVDILHLDAHPDIYDCFEGNTYSHASSFARIMEGGYARRLLQVGLRSITKEGREQGKRFGVEQYEMRTFSRDREKLENLKLGEGVKGVYVSVDVDCLDPAFAPGVSHIEPGGLSFRDVLNILQNLQGDVVAGDVVEFNPQRDTVDGMTAMVAAKLVRELSAKISK

>Traes_3AL_302AF461E.1 4565.Traes_3AL_302AF461E.1 Uncharacterized protein

MRGEKIGTLFHNEANVWDCSKEVTTREMAVAAKDCSRHLQNLSSEERKKILLDIAGALDANVDLIISENEADLAAAQDSGYEKSLVARMTLKAGKITSLAESIRAIADMEDPISHTLKKTEVAKDLVFEKMYCPLGVLLIIFESRPDALVQIAALAIRSGNGLLLKGGKEAMRSNTILHKVITSVIPDVVGKKLIGLVKSKDEIADLLKLDDVIDLVIPRGSNRLVSQIKAQTKIPVLGHADGICHVYIDKSADMDMAKRIVLDETLLVHKDLNKTEGLDDLLMELAKEGVVIYGGPVAHDTLKVPKVDSFHHEYSSMACTLEFVDDVQSAIDHINRYGSAHTDCIITTDKKSADTFLQQVDSAAVFHNASTRFCDGTRFGLGAEVGISTGRIHARGPVGVDGLLTTRCILRGSGQVVNGDKGVVYTHKDLPLQ

>Traes_3B_1E5C683B5.1 4565.Traes_3B_1E5C683B5.1 Pyrroline-5-carboxylate reductase ; Belongs to the pyrroline-5-carboxylate reductase family

ANGGDAFRLGFVGAGNLAESIARGVAASGVLPASAVRTAPHRRPERGAAFASLGATILASNAQVVDGSDVIVISVKPQIVKQVLVELKPLLSEEKLLVSIAAGIKMKDLQDWSGQRRIIRVMPNTPSAVGQAASVMCLGETATEKDENRVKSLFSAIGKVWTAEEKYFDAVTGLSGSGPAYIFLAIEAMADGGVAAGLPRDLALGLAAQTVLGAATMVSETGKHPGQLKDQVTSPAGTTIAGVHELEKGSFRGTLINAVVAATTRCRELSKN

>Traes_3B_C4683D0FA.2 4565.Traes_3B_C4683D0FA.2 Delta-1-pyrroline-5-carboxylate synthase; P5CS plays a key role in proline biosynthesis, leading to osmoregulation in plants; In the N-terminal section; belongs to the glutamate 5- kinase family

MGRGGIGGAVAAADLENSDSTRGFVRDVKRIVVKVGTAVVTGQNGRLAMGRLGALCEQVKELNFQGYEVILVTSGAVGVGRQRLKYRKLINSSFADLQNPQLDLDGKACAAVGQSGLMAIYDTLFSQLDVTSSQLLVTDRDFRDPSFGHQLRETVVSLLDLKVIPVFNENDAISTRRAPYEDSSGIFWDNDSLATLLAKELDADLLIMLSDVEGLYSGPPSDPQSKIIHTYINEKHGKLINFGEKSRVGRGGMQAKVAAAVTAASKGVPAVIASGFVTDSIIKIMRGEKIGTLFHNEANVWDCSKEVTTREMAVAAKDCSRHLQNLSSEERKKILLDIAGALDANVDLIISENEADLAAAQDSGYEKSLVARMTLKAGKITSLAESIRAIADMEDPISHTLKKTEVAKDLVFEKMYCPLGVLLIIFESRPDALVQQIAALAIRSGNGLLLKGGKEAMRSNTILHKVITSVIPDAVGKKLIGLVKSKDEIADLLKLDDVIDLVIPRGSNRLVSQIKAQTKIPVLGHADGICHVYIDKSADMDMAKRIVLDAKVDYPAACNAMETLLVHKDLNKTEGLDDLLMELAKEGVVIYGGPVAHDTLKVPKVDSFHHEYSSMACTLEFVDDVQSAIDHINRYGSAHTDCIITTDKKSADTFLQQVDSAAVFHNASTRFCDGTRFGLGAEVGISTGRIHARGPVGVDGLLTTRCILRGSGQVVNGDKGVVYTHKDLPLQ

>Traes_3DL_3E215D878.2 4565.Traes_3DL_3E215D878.2 Uncharacterized protein

MGRLGALCEQVKELNFQGYEVILVTSGAVGVGRQRLKYRKLINSSFADLQNPQLDLDGKACAAVGQSGLMAIYDTLFSQLDVTSSQLLVTDRDFRDPSFGHQLRETVVSLLDLKVIPVFNENDAISTRRAPYEDSSGIFWDNDSLATLLAKELDADLLIMLSDVEGLYSGPPSDPQSKIIHTYINEKHGKLINFGEKSRVGRGGMQAKVAAAVTAASKGVPAVIASGFVTDSIIKIMRGEKIGTLFHNEANVWDCSKEVTTREMAVAAKDCSRHLQNLSSEERKKILLDIAGALDANVDLIISENEADLAAAQDSGYEKSLVARMTLKAGKITSLAESIRAIADMEDPISHTLKKTEVAKDLVFEKMYCPLGVLLIIFESRPDALVQIAALAIRSGNGLLLKGGKEAMRSNTILHKVITSVIPDAVGKKLIGLVKSKDEIADLLKLDDVIDLVIPRGSNRLVSQIKAQTKIPVLGHADGICHVYIDKSADMDMAKRIVLDAKVDYPAACNAMETLLVHKDLNKTEGLDDLLMELAKEGVVIYGGPVAHDTLKVPKVDSFHHEYSSMACTLEFVDDVQSAIDHINRYGSAHTDCIITTDKKSADTFLQQVDSAAVFHNASTRFCDGTRFGLGAEVGISTGRIHARGPVGVDGLLTTRCILRGSGQVVNGDKGVVYTHKDLPLQ

>Traes_3DL_EB6A17449.1 4565.Traes_3DL_EB6A17449.1 Pyrroline-5-carboxylate reductase

MKDLQDWSGQRRIIRVMPNTPSAVGQAASVMCLGETATENDENRVKSLFSAIGKVWTAEEKYFDAVTGLSGSGPAYIFLAIEAMADGGVAAGLPRDLALGLAAQTVLGAATMVSETGKHPGQLKDQVTSPAGTTIAGVHELEKGSFRGTLINAVVAATTRCRELSKN

>Traes_4AL_30166AE64.1 4565.Traes_4AL_30166AE64.1 Uncharacterized protein

GDFDTCGLYADQLKRRCAEQFKEGVLGLEHLAAVDGLCEIVARGMGAAEGPRRYHINLSVFTSLPDMWAIEQLFPIIPIQRLQERPAV

>Traes_4AL_D98D91F71.1 4565.Traes_4AL_D98D91F71.1 Uncharacterized protein

KKKKCSPAVFLKKEKTALHLQPTGAAADARRHLQAARQVVAAARQASKQPTPAAGPRTSRRRPYSSSVVATSKLQGFVGMGNLAESIACGVAASGVLSASAIRTTVHRHPERCAAFASLGATILASNAQVVEDSDVIVISIKPQIVKKVLVELKPLLSEEKLLVSITAAIKNERFTDWPVYSGFTELLKL

>Traes_4BL_E4445BC35.1 4565.Traes_4BL_E4445BC35.1 Cyclin-dependent kinases regulatory subunit

ARRHVVLPPEVAKLLPKNRLLAENEWRALGVQQSRGWVHYAVHRPEPHIMLFRRPLNYQQQQDAAAAAAAQMMPK

>Traes_4DL_C2A1EEB2C.1 4565.Traes_4DL_C2A1EEB2C.1 Cyclin-dependent kinases regulatory subunit; Binds to the catalytic subunit of the cyclin dependent kinases and is essential for their biological function

MGQIQYSEKYFDDTFEYRHVVLPPEVAKLLPKNRLLAENEWRALGVQQSRGWVHYAVHRPEPHIMLFRRPLNYQQQQDAAAAAAAQMMPK

>Traes_5AL_8349DE248.1 4565.Traes_5AL_8349DE248.1 Uncharacterized protein

MVFSKGEGSHILDPEGNKYIDFLSAYSAVNQGHCHPKVLRALIEQAERLTLSSRAFYNDKFPVFAQYLTSMFGYDMMLPMNTGAEGVETAIKLARKWGYEKKNIPKNEALIVSCCGCFHGRTLGVISMSCDNDATRGFGPLVPGHLKVDFGDIDGLEKIFKEHGDRICGFLFEPIQGEAGVIIPPDGYLKAVRDLCSRHNILMIDDEIQTGIARTGKMLACDWEGVRPDMVILGKALGAGVVPVSAVLADKDIMLCIKPGEHGSTFGGNPLASAVAIASLKVVKDEGLVERAAELGQEFRDQLRKVQQKFPDIIREIRGRGLLNAVDLSSKALYPASAYDICIKLKERGILAKPTHDTIIRLAPPISISPEELTEASKALSDVLEHDLPQLQKQIKKPESEAKTPVCDRCGRDL

>Traes_5BL_1D3F1BCC2.1 4565.Traes_5BL_1D3F1BCC2.1 Uncharacterized protein; Belongs to the class-III pyridoxal-phosphate-dependent aminotransferase family

MAAIISRRGAARALALAMARRGMCSAPAPAAALSSEELIRMEQDCSAHNYHPIPMVFSKGEGSHILDPEGNKYIDFLSAYSAVNQGHCHPKVLRALIEQAERLTLSSRAFYNDKFPVFAQYLTSMFGYDMMLPMNTGAEGVETAIKLARKWGYEKKNIPKNEALIVSCCGCFHGRTLGVISMSCDNDATRGFGPLVPGHLKVDFGDIDGLEKIFKEHGDRICGFLFEPIQGEAGVIIPPDGYLKAVRDLCSRHNILMIDDEIQTGIARTGKMLACDWEDVRPDMVILGKALGAGVVPVSAVLADKDIMLCIKPGEHGSTFGGNPLASAVAIASLKVVKDEGLVERAAELGQEFRDQLQKVQQKFPRIIREIRGRGLLNAVDLSSKALYPASAYDICIKLKERGILAKPTHDTIIRLAPPISISPEELAEASKALSDVLEHDLPQLQKQIKKPDSEAKIPVCDRCGRDL

>Traes_5BL_6E095245A.1 4565.Traes_5BL_6E095245A.1 Arginine decarboxylase; Belongs to the Orn/Lys/Arg decarboxylase class-II family. SpeA subfamily

MMDPATAYLLDELTDDCRSEYRNLMASAVRGDFETCGLYADQLKRHCAEQFKEGVLGLEHLAAVDGLCEIVARGMGTAEGPRRYHINLSVFTSLPDMWAIEQLFPIIPIQRLQERPAVDGVLSDLTCDSDGKVDQFIGGRSSLPLRSNFVFLTLFANKIGI

>Traes_5DL_AC1F44221.1 4565.Traes_5DL_AC1F44221.1 Uncharacterized protein; Belongs to the class-III pyridoxal-phosphate-dependent aminotransferase family

MVFSKGEGSHILDPEGNKYIDFLSAYSAVNQGHCHPKVLRALIEQAERLTLSSRAFYNDKFPVFAEYLTSMFGYDMMLPMNTGAEGVETAIKLARKWGYEKKNIPKNEALIVSCCGCFHGRTLGVISMSCDNDATRGFGPLVPGHLKVDFGDIDGLEKIFKEHGDRICGFLFEPIQGEAGVIIPPDGYLKAVRDLCSRHNILMIDDEIQTGIARTGKMLACDWEDVRPDMVILGKALGAGVVPVSAVLADKDIMLCIKPGEHGSTFGGNPLASAVAVASLKVVKDEGLVERAAELGQEFRDQLQKVQQKFPHIIREIRGRGLLNAVDLCSKALYPASAYDICIKLKERGILAKPTHDTIIRLAPPLSISSEELAEASKALSDVLEHDLPQLQKQIKKPESEAKTPVCDRCGRDL

>Traes_6DS_A879508C3.1 4565.Traes_6DS_A879508C3.1 Uncharacterized protein

MAMAMRRAAALGARHILAASVSASSRVASRRHMGSVDAGAAMEKIRAAGLLRTRGLIGGKWVDAYDGKTIEVQNPATGEVLANVACMGNRETADAITSANTTFYTWSKLTASERSKALRKWHDLLMSHKEELALLMTLEQGKPMKEALGEVNYGASFIEFFAEEAKRVYGDIIPPTLADRRLLVLKQPIGVVGAITPWNFPLAMITRKVGPALACGCTVVVKPSEFTPLTALAAADLALQAGIPAGALNVVMGNAPEIGDELMQSMQVRKITFTGSTAVGKKLMAGSANTVKKVSLELGGNAPCIVFDDADIDVAVKGSLAAKFRNSGQTCVCANRILVQEGIYEKFASAFVKAVQSLQVGNGLEESTSQGPLINEAAVQKGANIMLGGKRHSLGMSFYEPTVVGNVSNDMLLFSEEVFGPVAPLIPFKTEEEAIHLANDTNAGLAAYMFTKNIARSWRVSEALEYGLVGVNEGLISTEVAPFGGVKQSGLGREGSKYGMDDYLEIKYVCMGNLG
